# Type IV collagen drives alveolar epithelial–endothelial association and the morphogenetic movements of septation

**DOI:** 10.1186/s12915-016-0281-2

**Published:** 2016-07-13

**Authors:** Maria Loscertales, Fotini Nicolaou, Marion Jeanne, Mauro Longoni, Douglas B. Gould, Yunwei Sun, Faouzi I. Maalouf, Nandor Nagy, Patricia K. Donahoe

**Affiliations:** The Pediatric Surgical Research Laboratories, Massachusetts General Hospital, Boston, MA 02114 USA; Department of Surgery, Harvard Medical School, Boston, MA 02115 USA; Departments of Ophthalmology and Anatomy, Institute for Human Genetics, University of California, San Francisco, School of Medicine, San Francisco, CA 94143 USA; Department of Human Anatomy, Histology and Embryology, Faculty of Medicine, Semmelweis University, Budapest, 1094 Hungary; Broad Institute of MIT and Harvard, Cambridge, MA 02142 USA

**Keywords:** Type IV collagen, Basement membrane, Blood–gas barrier, Alveolar development, Lung epithelium, Lung vasculature, Alveolar myofibroblast migration and differentiation

## Abstract

**Background:**

Type IV collagen is the main component of the basement membrane that gives strength to the blood–gas barrier (BGB). In mammals, the formation of a mature BGB occurs primarily after birth during alveologenesis and requires the formation of septa from the walls of the saccule. In contrast, in avians, the formation of the BGB occurs rapidly and prior to hatching. Mutation in basement membrane components results in an abnormal alveolar phenotype; however, the specific role of type IV collagen in regulating alveologenesis remains unknown.

**Results:**

We have performed a microarray expression analysis in late chick lung development and found that *COL4A1* and *COL4A2* were among the most significantly upregulated genes during the formation of the avian BGB. Using mouse models, we discovered that mutations in murine *Col4a1* and *Col4a2* genes affected the balance between lung epithelial progenitors and differentiated cells. Mutations in *Col4a1* derived from the vascular component were sufficient to cause defects in vascular development and the BGB. We also show that Col4a1 and Col4a2 mutants displayed disrupted myofibroblast proliferation, differentiation and migration. Lastly, we revealed that addition of type IV collagen protein induced myofibroblast proliferation and migration in monolayer culture and increased the formation of mesenchymal–epithelial septal-like structures in co-culture.

**Conclusions:**

Our study showed that type IV collagen and, therefore the basement membrane, play fundamental roles in coordinating alveolar morphogenesis. In addition to its role in the formation of epithelium and vasculature, type IV collagen appears to be key for alveolar myofibroblast development by inducing their proliferation, differentiation and migration throughout the developing septum.

**Electronic supplementary material:**

The online version of this article (doi:10.1186/s12915-016-0281-2) contains supplementary material, which is available to authorized users.

## Background

The lung is a complex, precisely structured organ in which the vascular network is intimately associated with epithelial-lined tubes and sacs for the prime purpose of gas exchange. In the developing lung, the specific temporal-spatial interactions between mesenchymal and epithelial cells are carefully orchestrated to permit the gradual establishment of an effective blood–gas barrier (BGB) [1, 2]. The formation of the mammalian respiratory unit is initiated during the transition between the pseudoglandular and canalicular/saccular stages and is characterized by coordinated proliferation and differentiation of epithelial and mesenchymal cells. During the saccular stage, the distal tips of the pulmonary airways dilate and primary septa form. Type I (forming the internal epithelial (alveolar) layer) and type II pneumocytes continue differentiating from primordial bronchoalveolar cells and the interstitial mesenchyme becomes thinner [3, 4]. Later, as alveolarization progresses, secondary septa develop and the microvascular network matures from a double to a single capillary network [2, 5, 6].

**Fig. 1:**
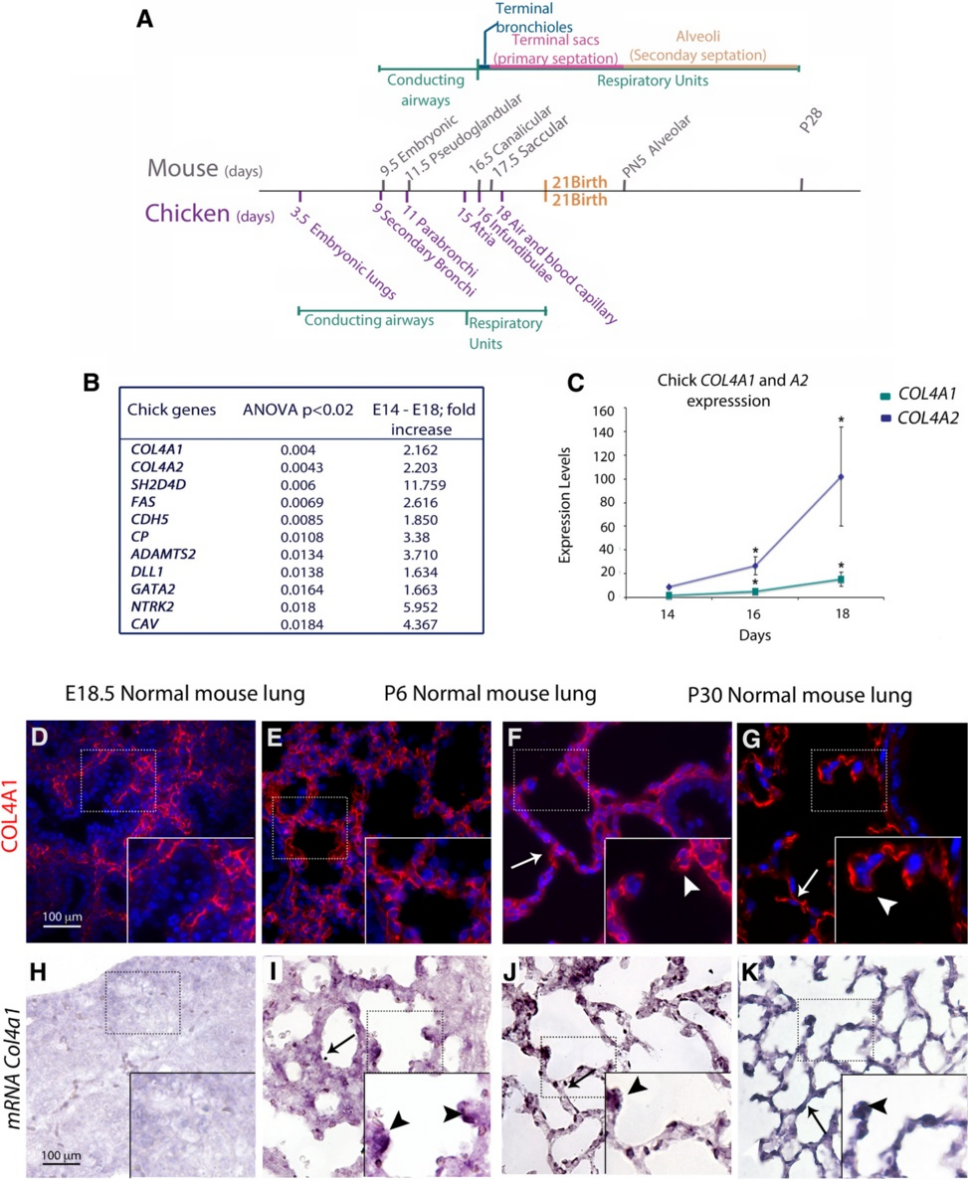
Lung development timeline and type IV collagen expression in the chicken and the mouse. **a** Mouse and chicken lung development comparative timeline. **b** Microarray analysis shows that vascular related genes, among which are *Col4a1* and *Col4a2*, show the highest significance in late chick lung development. **c** Real-time PCR shows differential expression of *Col4a1* (blue) and *Col4a2* (green) between E16 and E18 in chick lungs. *Col4a1* and *Col4a2* expression increases at E16 and E18, and is statistically significant (Wilcoxon rank-sum test *P* < 0.05) when compared to E14. Chicken *G6PDH* was used as a normalizer. **d**–**i** Type IV collagen protein and mRNA expression in the lung. **d**–*g* COL4A1 protein is found throughout the lung interstitium and epithelium (arrows) in the prenatal (E18.5) and postnatal (P6, P30) lungs but mainly in the interstitium at E16.5. At P6 and P30 murine COL4A1 protein is found at the tips of secondary septa (arrowhead in FGF). **h**–k *Col4a1* mRNA is almost undetectable by in situ hybridization at E16.5 (**h**) and is later found in a patchy distribution throughout the interstitium (arrows in **i**–**k**) with high expression at the tips of primary and secondary septa *(*arrowheads). Scale bar = 100 μm and applies to **d**–**i**

The essential components of the BGB were established early in evolution and are conserved among vertebrates. Only mammals and birds have a complete separation of pulmonary and systemic circulations [7]. Despite anatomical differences, the lungs of both species are functional equivalents, so the chick can serve as an effective experimental surrogate for the formation of the BGB in mammals (Fig. 1a). In contrast to later postnatal development in mammals, the pulmonary BGB in chick develops primarily in ovo prior to hatching and is characterized by a massive increase in air and blood capillaries, which develop with a progressive reduction of the lung interstitium [8, 9].

Type IV collagen is the major component of the BGB epithelial and endothelial basement membranes and is responsible for its strength [7, 10]. The most widely distributed form of collagen IV is the isoform [a1(IV)]2,a2(IV) [11–13]. Homozygous mutations of *Col4a1* and *Col4a2* (*Col4a1*^*–/–*^; *Col4a2*^*–/–*^) are lethal after mid-embryogenesis (E10.5–E11.5) because of impaired basement membrane stability [10]. Heterozygous mutants developed porencephaly with severe perinatal cerebral hemorrhage, along with ocular and renal abnormalities; pups were also often observed to be cyanotic with respiratory distress, dying soon after birth [14, 15]. Most *Col4a1*^*+/Δex41*^ heterozygous mouse mutants with deletion of exon 41 exhibited compact lungs with little or no detectable terminal air spaces [15]. *Col4a1*^*+/Δex41*^ mutations had been shown to cause an increase of intracellular accumulation and a decrease of extracellular collagen, and the decrease of extracellular type IV collagen might contribute to the abnormal angiogenesis observed in *Col4a1*^*+/Δex41*^ mutants [16, 17]. Abnormalities in vascular development are also observed in *Col4a1*^*–/–*^; *Col4a2*^*–/–*^ mutants [10].

Multiple paracrine signals between epithelium, endothelium and mesenchymal stroma direct alveolar development and need to be coordinated to ensure the proper formation of the BGB at birth [18]. The pulmonary vasculature is essential for alveoli formation, which is disrupted when angiogenesis is inhibited [19–23]. Equally important are the alveolar myofibroblasts, believed to direct alveolar septal formation by controlling elastin synthesis [24, 25]. During alveologenesis, platelet-derived growth factor receptor alpha (PDGFRα) expressing myofibroblast progenitors differentiate into alpha smooth muscle actin (α-SMA) alveolar myofibroblasts which are anchored onto the basement membrane [26–29]. Mice with defective myofibroblast differentiation and proliferation reveal phenotypes of impaired alveolar development [30–32].

Defective alveolarization is observed in immature lungs from babies born prematurely or with lung hypoplasia, and in chronic lung disease [23, 33–41]. Lung immaturity is the consequence of the arrest of lung development between the saccular and the alveolar stages, whose molecular mechanisms remain unclear despite their critical role for the formation of the gas exchange unit. Taking advantage of the rapid avian pulmonary development, we studied gene expression trends occurring mid to late in BGB formation using microarrays with *COL4A1* and *COL4A2* among the most upregulated genes. Although mutations in several basement membrane components, including *Col4a1*^*+/Δex41*^, have been reported to result in little or no detectable terminal air spaces [15, 42–44], very little is known about the role of the basement membrane or collagens in alveologenesis. We found that *Col4a1* and *Col4a2* mutations caused defects in epithelial, endothelial and mesenchymal alveolar patterning that result in an aberrant BGB combined with defective elastin deposition and septa formation. *Col4a1*^*+/Δex41*^ conditional expression in vascular cells was sufficient to cause a disorganized BGB. Finally, we determined that type IV collagen directs myofibroblast proliferation and migration, and the formation of septal-like formations in vitro. Type IV collagen appears to control the epithelial, endothelial and myofibroblast components of the alveolar unit, thereby permitting adequate gas exchange.

## Results

### Microarray analysis identifies high COL4A1 and COL4A2 expression at the time of chick BGB formation

Taking advantage of the more rapid in ovo formation of the avian BGB respiratory units (Fig. 1a), we isolated RNA from *Gallus gallus* embryonic lungs at embryonic days (E)14, 15, 16, and 18, in triplicate. The microarray data analysis showed 892 significantly differentially expressed transcripts (*P* < 0.05), corresponding to 551 unique mouse orthologs listed by Ensembl BioMart. The differentially regulated genes between E14 and E18 were annotated by the Mouse Genome Informatics (MGI) VisuaL Annotation Display (Vlad) tool. The most significantly enriched Gene Ontology (GO) categories were angiogenesis (GO: 0001525) and extracellular matrix (GO: 0044421, GO: 0044421, GO: 0005615). Vascular development was among the most prominent processes at this stage (Additional file 1: Figure S1A–C). Between E14 and E18, there were 24 vascular up-regulated genes, 11 of which had ANOVA *P* < 0.02 (Fig. 1b). Interestingly, the up-regulated genes *COL4A1* and *COL4A2* were present across every significant GO category identified (Additional file 1: Figure S1B). Real time-qPCR confirmed the increase of *COL4A1* and *COL4A2* in the chick lung during gestation (Fig. 1c).

### Col4a1 and Col4a2 are essential in the mouse for saccular and alveolar growth

We used a number of mouse genetic models to study the role of type IV collagen during lung organogenesis, including three distinct mutations from an allelic series of *Col4a1* and *Col4a2* mutations and a *Col4a1* conditional allele [17, 45–47]. Mutations from the allelic series include a point mutation in *Col4a1* (*Col4a1*^*G394V*^), a point mutation in *Col4a2* (*Col4a2*^*G646D*^) and a splice site mutation in *Col4a1* that results in a deletion of exon 41 (*Col4a1*^*Δex41*^). In *R26-Cre*^*ER*^*; Col4a1*^*+/Flex41*^ and *Tie2-Cre; Col4a1*^*+/Flex41*^ conditional mice, the mutation was generated by flanking exon 41 with *LoxP* sites and therefore recreates the *Col4a1*^*Δex41*^ mutation in a CRE-dependent manner (called *Col4a1*^*Flex41*^) [17]. Besides having different mutations, all of the strains are otherwise genetically identical.

**Fig. 2:**
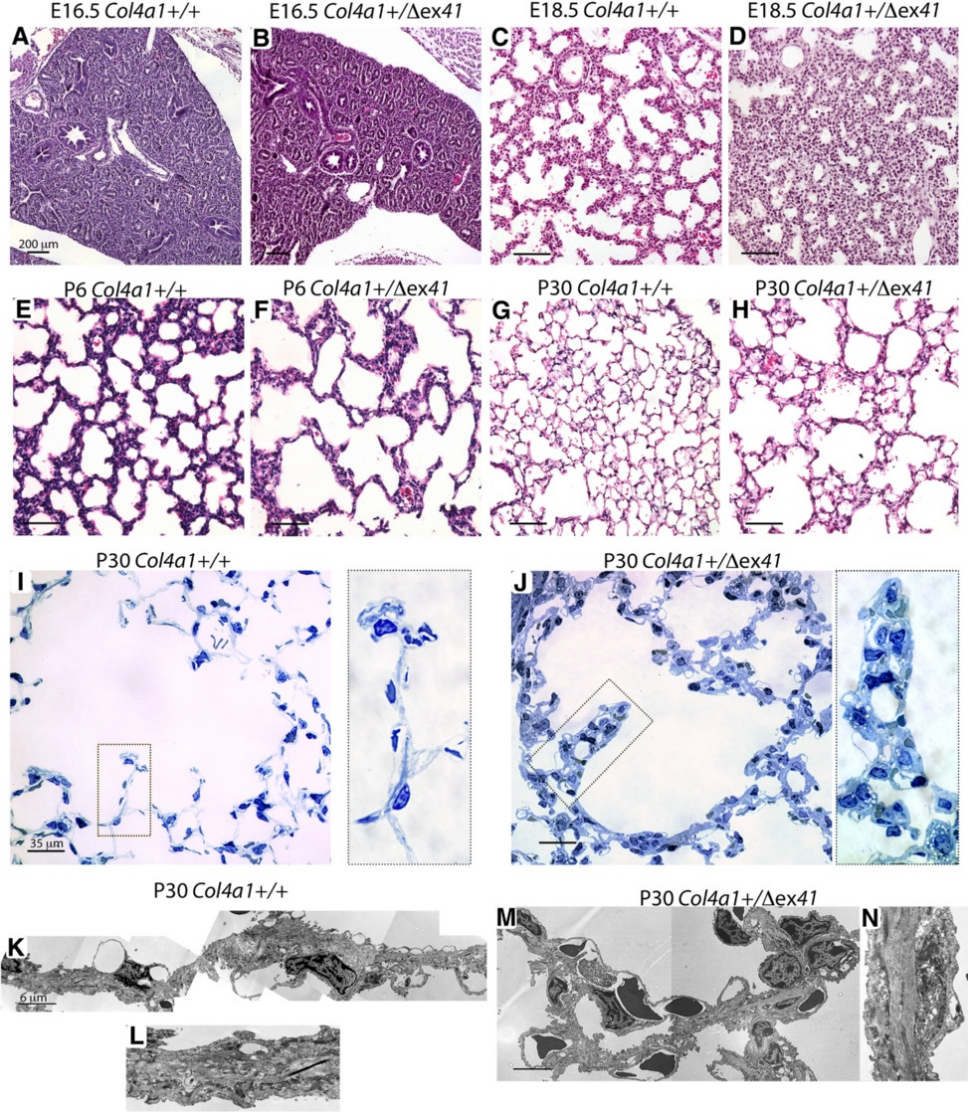
Histological examination of *Col4a1* ^*+/Δex41*^. **a**, **b** Hematoxylin and eosin staining of normal and *Col4a1* mutant lungs at E16.5 shows typical pseudoglandular structures. **c** At E18.5, normal lungs are composed of dilated distal tubules surrounded by relatively thin mesenchyme with arising primary septa, which are characteristic of the saccular stage. **d** *Col4a1* ^*+/Δex41*^ lungs have fewer dilated tubules, thick interstitium, and fewer and shorter primary septa. **e** Normal alveolar and development at P6, showing secondary septa. **f** P6 *Col4a1* ^*+/Δex41*^ have fewer fully developed alveoli. **g** At P30, normal lungs show thin secondary septa and interstitium. **h** *Col4a1* ^*+/Δex41*^ mutant lungs in surviving mice display much fewer secondary septa and a thicker interstitium. **i** Toluidine blue staining of *Col4a1* ^*+/+*^ semi-thin lung sections show capillaries (arrowheads). **j** In *Col4a1* ^*+/Δex41*^, the septa have an increase of interstitial blood capillaries (arrowhead) and cells with lipid content (arrows). **k**–**n** Electron microscopy of normal and *Col4a1* ^*+/Δex41*^ lung septa at P30. **m** *Col4a1* ^*+/Δex41*^ septum showing capillaries (arrowheads), type II pneumocytes (arrows) and elastin fibers (red arrows). Scale bars = 200 μm in **a**–**d**, 35 μm in **i**, **j**, and 6 μm in **k**, **l**

In normal mouse lungs at E16.5, E18.5, postnatal day (P)6 and P30, type IV collagen is expressed in the interstitium. COL4A1 localization in the epithelial basement membrane at the branching tips at E16.5 is almost undetectable (Fig. 1d), but it is found in proximal epithelium and also at the tip of secondary septa (Fig. 1d–f). *Col4a1* mRNA expression was very week at epithelial tips and surrounding mesenchyme at E16.5 (Fig. 1h), but later, at E18.5, P6, and P30, it was clearly found in the lung interstitium and at the tips of the developing septa (Fig. 1g–i). Hematoxylin and eosin (H&E) staining of the lungs of *Col4a1*^*+/Δex41*^ mutants at E16.5 showed no clear difference in branching morphogenesis compared with wild type lungs (Fig. 2a, b). Later, at E18.5 (saccular stage), *Col4a1*^*+/Δex41*^, *Col4a1*^*+/G394V*^ and *Col4a2*^*+/G646D*^ displayed lung hypercellularity and thickened interstitia. Moreover, the tips of the distal airways in lungs from mutant mice failed to form saccules and primary septa (Fig. 2c, d and Additional file 2: Figure S2A, B). Surviving *Col4a1*^*+/Δex41*^ mutants have reduced viability [14]; however, those that survive to P6 and 1 month (P30) have an emphysematous-like phenotype with simplified alveolarization and an overall decrease in the number of secondary septa (Fig. 2e–h). Toluidine blue staining of lungs from *Col4a1*^*+/Δex41*^ mice also showed alveolar simplification with thick septa (Fig. 2i, j). Closer examination of *Col4a1*^*+/Δex41*^ lungs showed an increase of blood capillaries and cells with lipid content (Fig. 2j). Both the inducible *R26-Cre*^*ER*^*; Col4a1*^*+/Flex41*^ and vascular endothelial restricted *Tie2-Cre; Col4a1*^*+/Flex41*^ conditional mutants also showed simplified alveoli formation at P30 (Additional file 3: Figure S3A, B) with *R26-Cre*^*ER*^*; Col4a1*^*+/Flex41*^ lungs having a phenotype similar to *Col4a1*^*+/Δex41*^ lungs. *Tie2-Cre; Col4a1*^*+/Flex41*^ conditional lungs have numerous blood capillaries, but accumulation of cells with lipid content in the septa is not observed (Additional file 3: Figure S3B, D). Electron microscopy of *Col4a1*^*+/Δex41*^ also showed increased capillaries and thick interstitium in septa (Fig. 2k, l). Lung septa also appear to display an excess of elastin fibers (Fig. 2l, n).

### Type IV collagen in the mouse regulates distal epithelial cell proliferation and differentiation during saccular formation

**Fig. 3:**
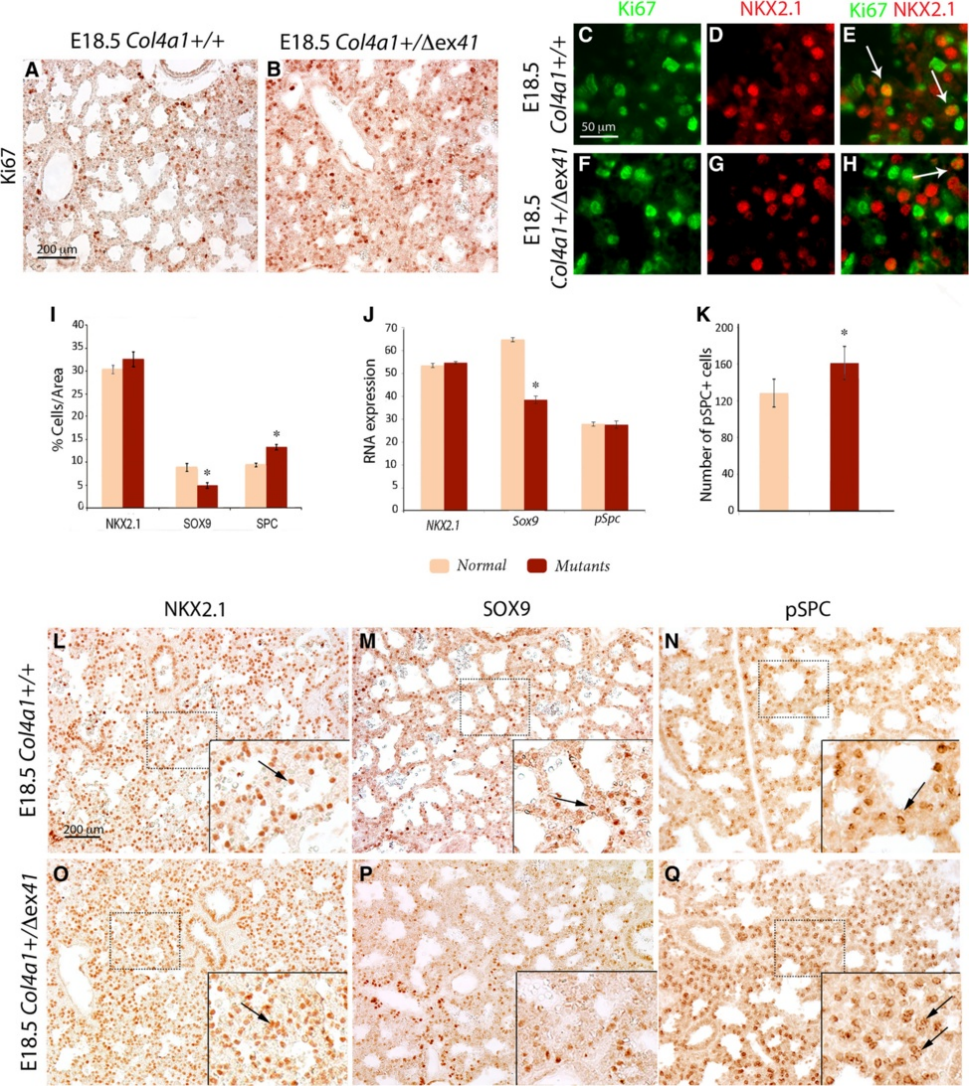
Decreased epithelial progenitors and increased type II pneumocytes in *Col4a1* ^*+/Δex41*^. **a**–**j** Epithelial proliferation and differentiation were evaluated by staining with Ki67, NKX2.1, SOX9, and pSPC. **a**, **b** Overall proliferation evaluated by Ki67 is increased in *Col4a1* ^*+/*Δex41^ lungs. **c**–**h** Double immunohistochemistry for Ki67 and NKX2.1 shows slightly active epithelial proliferation in NKX2.1 cells (arrows) in normal (**c**–**e**) or *Col4a1* ^*+/Δex41*^ lungs at E18.5 (**f**–**h**). **l** The bar charts show the percentage NKX2.1, SOX9 progenitors and pSPC^+^ cells over the total distal area of the lung of *Col4a1* and *Col4a2* mutants and wild type mice. *Col4a1* ^*+/Δex41*^ mutants have a statistically significant decrease of SOX9 cells and an increase of pSPC type II pneumocytes, while NKX2.1^+^ cells are unchanged compared with normal lungs at E18.5. **j** Real-time PCR of *Nkx2.1*, *Sox9* and *pSpc*. Only *Sox9* mRNA expression is decreased in *Col4a1* ^*+/Δex41*^. *Gapdh* was used as a normalizer. **k** The number of pSPC^+^ cells at P30 is increased in mutant lungs. **l**, **o** NKX2.1, **m**, **p** SOX9 and **n**, **q** pSPC localization in normal lungs displays a scattered pattern around the saccular walls (arrows), while in *Col4a1* ^*+/Δex41*^ mutants NKX2.1 and pSPC are clustered together (arrows). Scale bars = 200 μm in **a**, **b**, 50 μm in **c**–**h**, and 200 μm in **l**–**q**

**Fig. 4:**
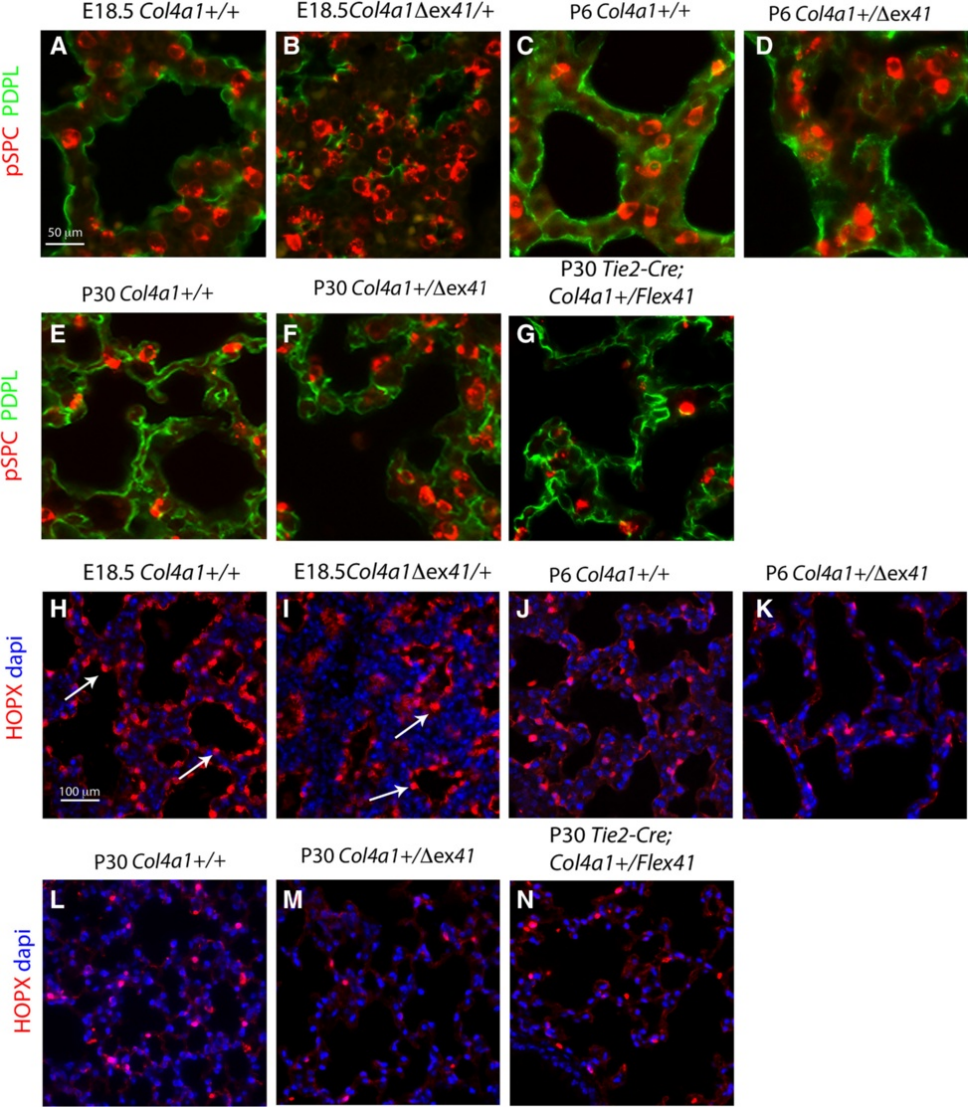
Abnormal alveolar epithelium and decreased type I pneumocytes in *Col4a1* ^*+/Δex41*^. **a**–**g** co-staining of pSPC and podoplanin (PDPL). **a**, **c**, **e** PDPL normally lines the majority of the internal saccular surface and alveoli. **b** At E18.5, *Col4a1* ^*+/Δex41*^ PDPL lined the collapsed saccules, which are surrounded by clusters of pSPC cells. **d**, **f** At P6 and P30, the *Col4a1* ^*+/Δex41*^ alveolar epithelium is disorganized, as PDPL and pSPC staining shows. **g** *Tie2-Cre; Col4a1* ^*+/Flex41*^ mice pSPC cell clustering was absent. **h**–**n** HOPX staining in the nucleus and cytosol of type I pneumocytes. (**i**, **k**, **m**) *Col4a1* ^*+/Δex41*^ lungs display fewer nuclear HOPX^+^ cells (arrows), more prominently at P6 and P30. **n** *Tie2-Cre; Col4a1* ^*+/Flex41*^ lungs show similar staining to normal lungs. Scale bars = 50 μm in **a**–**g** and 200 μm in **h**–**n**

To investigate whether the lung hypercellularity is the result of abnormal distal epithelial cell proliferation, we co-stained E18.5 lungs with the proliferative Ki67 and the lung epithelial lineage factor NK2 homeobox 1 (NKX2.1) markers. Ki67 at E18.5 showed an overall increase of cell proliferation (Fig. 3a, b) with little or no epithelial proliferation, as indicated by NKX2.1 and Ki67 co-localization (Fig. 3c–h; and 48). Next, we studied distal epithelial differentiation by staining with NKX2.1, SRY-homeobox 9 (SOX9) and pro-surfactant protein C (pSPC), and discovered a statistically significant decrease of SOX9^+^ progenitor cells (Student’s *t* test *P* < 0.05; Fig. 3i) and an increase of pSPC^+^ type II pneumocytes (*P* < 0.05; Fig. 3i). Real-time PCR of *Col4a1*^*+/Δex41*^ lungs confirmed decrease of mRNA expression of *Sox9* (Wilcoxon rank-sum test *P* < 0.05) but not an increase in *pSPC* mRNA (Fig. 3j). After alveolarization, an increase in pSPC^+^ cells was still observed (Fig. 3k). Morphologically, *Col4a1*^*+/Δex41*^, *Col4a1*^*+/G394V*^ and *Col4a2*^*+/*G646D^ had a patchy distribution with clustering of SOX9^+^, NKX2.1^+^ and pSPC^+^ cells versus normal lungs (Fig. 3l–q and Additional file 2: Figure S2C–G). At P6 and P30, heterozygous *Col4a1*^*+/Δex41*^ and *R26-Cre*^*ER*^*; Col4a1*^*+/Flex41*^ mice still displayed an increase of clustered pSPC^+^ type II pneumocytes (Additional file 4: Figure S4D, H, I) that was not observed in endothelium restricted *Tie2-Cre; Col4a1*^*+/Flex41*^ mice or in normal siblings (Additional file 4: Figure S4A, G, J). When, we co-stained *Col4a1*^*+/Δex41*^ lung sections with pSPC and the type I pneumocyte marker podoplanin (PDPL), we found that clusters of pSPC^+^ cells grouped around a fragmented PDPL at E18 and P6 (Fig. 4a–d). Later, at P30, the alveolar epithelium showed disorganization (Fig. 4e, f). This disorganization was observed in P30 *R26-Cre*^*ER*^*; Col4a1*^*+/Flex41*^ (Additional file 4: Figure S3E) but not obvious in *Tie2-Cre; Col4a1*^*+/Flex41*^ (Fig. 4g).

To more clearly show abnormalities in the development of type I pneumocytes, we also stained lung sections with the HOP homeobox (HOPX) antibody, which is specific for the nuclei and cytoplasm of type I pneumocytes [48]. Our data showed a decrease in the number of HOPX^+^ cells in *Col4a1*^*+/Δex41*^ at E18.5, P6, and P30 (Fig. 4h–m). P30 *Tie2-Cre; Col4a1*^*+/Flex41*^ lungs HOPX expression was similar to normal lungs (Fig. 4n) but it was reduced in *R26-Cre*^*ER*^*; Col4a1*^*+/Flex41*^ at P30 (Additional file 3: Figure S3F). When *Col4a1*^*+/Δex41*^ mice were analyzed before the saccular stage, at E16.5, no apparent differences in cellular proliferation, epithelial and mesenchyme markers were observed (Additional file 5: Figure S5A–L).

### Vascular type IV collagen is necessary for normal of epithelial and endothelial association

**Fig. 5:**
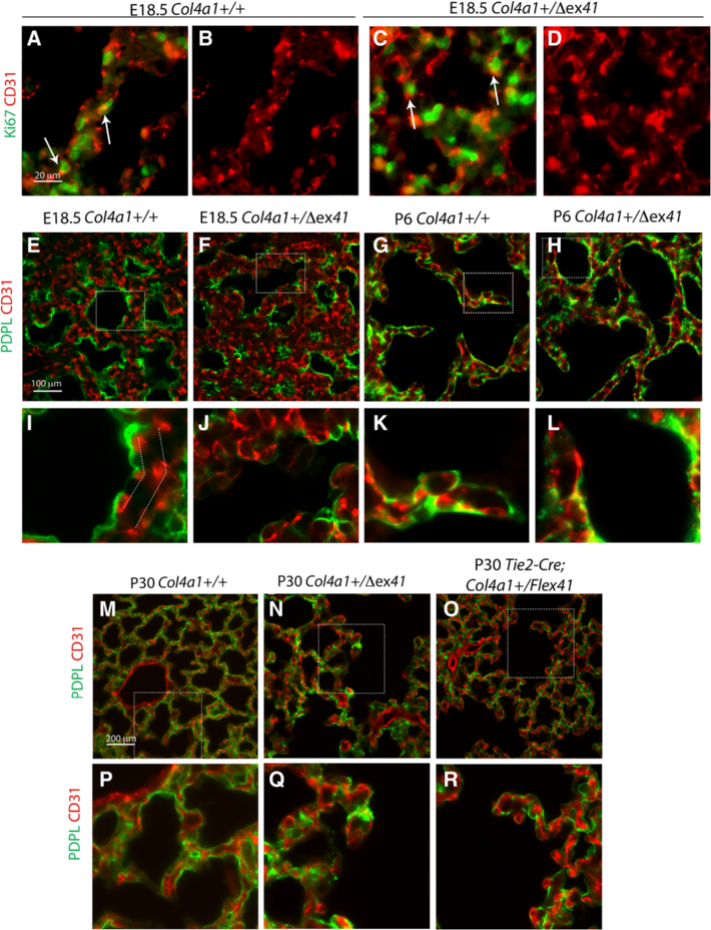
Abnormal microvasculature and endothelial–epithelial association in *Col4a1* ^*+/Δex41*^ mice. **a**–**d** Co-staining of Ki67 and CD31 shows active endothelial proliferation (arrows) at E18.5. **e** Endothelial cells stained with CD31 exhibit the characteristic double capillary layer of the saccular stage (**i**). **f** In contrast, *Col4a1* ^*+/Δex41*^ vascular plexus is disorganized within the interstitium and the alveolar epithelium marked with PDPL (**j**). Co-localization of PDPL and CD31 shows abnormal development of the blood–gas barrier in *Col4a1* ^*+/Δex41*^ at P6 (**g**, **h**, **k**, **l**) and P30 (**m**, **n**, **p**, **q**). **o**, **r** *Tie2-Cre; Col4a1* ^*+/Flex41*^ mice also show a disorganized endothelium–epithelium association. Scale bars = 20 μm in **a**–**d**, and 100 μm in **e**–**h** and **m**–**o**

To assess pulmonary microvasculature development in type IV collagen mutants, we stained endothelial cells for Cluster of Differentiation 31 (CD31) at E18.5, P6 and P30. First, we co-stained with CD31 and Ki67 at E18.5, which confirmed active endothelial proliferation in both normal and mutant lungs (Fig. 5a–d). At the saccular stage (E18.5), normal CD31 staining showed an organized double layer of endothelial cells (Fig. 5i) forming a lung vascular plexus that develop into a single layer (Fig. 5p) in close proximity to the alveolar epithelial type I pneumocytes during alveolar maturation (Fig. 5e, i, g, k, m, p). At E18.5, *Col4a1* and *Col4a2* mutants showed a tortuous vascular plexus spreading throughout the lung interstitium (Fig. 5f, j and Additional file 6: Figure S6A, B) which was exacerbated at P6 (Fig. 5g, h) and at the end of alveolarization (P30; Fig. 5n, q). Epithelial–endothelial disorganization was also observed in *Tie2-Cre; Col4a1*^*+/*Flex*41*^ (Fig. 5o, r) and in the postnatal-induced *R26-Cre*^*ER*^*; Col4a1*^*+/Flex41*^ (Additional file 5: Figure S5C, D) mice, indicating that loss of endothelial-derived type IV collagen is sufficient to disrupt the endothelial–epithelial association.

### Type IV collagen regulates myofibroblast proliferation and migration

**Fig. 6:**
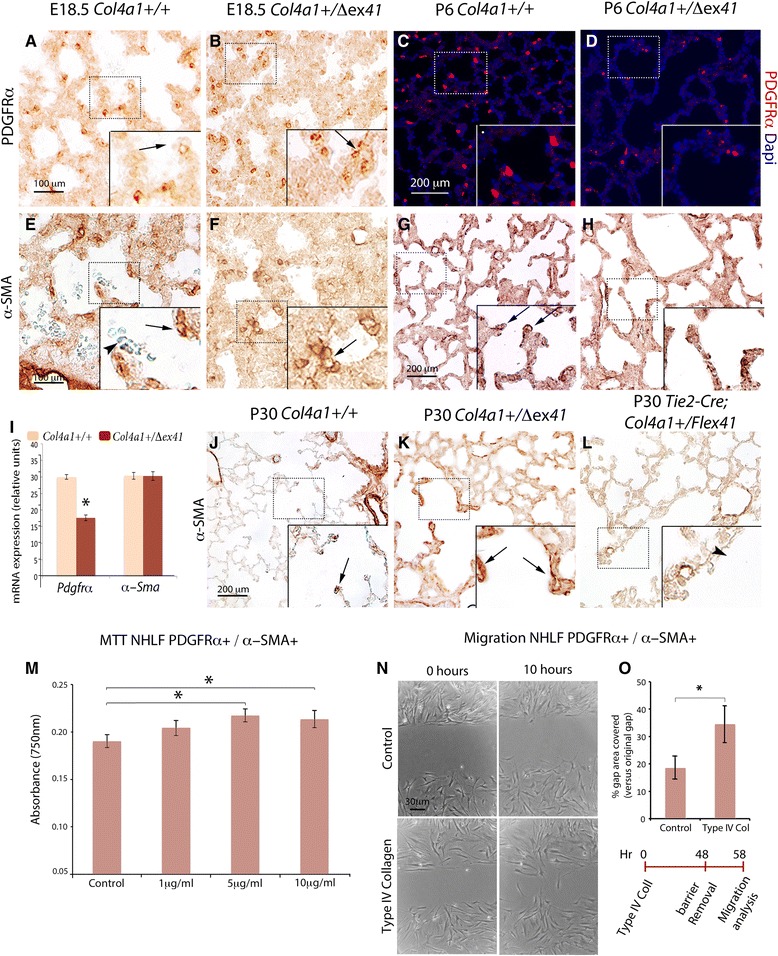
Abnormal development of alveolar myofibroblast in Col4a mutants. **a** Myofibroblast progenitors positive for PDGFRα are normally scattered in the alveolar walls and at the tips of primitive septa at E18.5 (arrows). **b** In *Col4a1* ^*+/Δex41*^ lungs, PDGFRα myofibroblast progenitors cluster in a patchy distribution (arrow). **c**, **d** In P6 *Col4a1* ^*+/Δex41*^, the number of PDGFRα^+^ is reduced. **e**, **f** α-SMA shows a decrease of differentiated alveolar myofibroblasts at the primary septa and a patchy distribution in *Col4a1* ^*+/Δex41*^ (arrow) when compared with normal lungs at E18.5 (arrow). Arrowheads in **c** point to red blood cells. **g** At P6, some α-SMA^+^ cells normally localize at the tips of developing septa (arrow). **h** *Col4a1* ^*+/Δex41*^ mutants display a decrease in septal and interstitial α-SMA^+^ cells. **i** Real-time PCR at E18.5 shows statistically significant decrease in *Pdgfrα* mRNA (Wilcoxon ran-sum test *P* < 0.05) but not in *α-Sma* mRNA. *Gapdh* was used as a normalizer. **j** At P30, α-SMA is normally localized at the tips of septa in the alveolar ducts (arrow). **k** *Col4a1* ^*+/Δex41*^ lungs have elongated α-SMA cells in alveolar septa (arrows). **l** These elongated α-SMA cells are not observed in the vascular conditional *Tie2Cre; Col4a1* ^*+/Flex41*^ mice, where α-SMA^+^ cells are found only in the vasculature (arrowhead). **m**–**o** Cell proliferation and migration of α-SMA^+^ NHLFs treated with type IV collagen protein. **m** Viability assay of α-SMA^+^ NHLFs treated with human type IV collagen are measured by absorbance, and show a significant increase in cell proliferation after 2 days of treatment with 5 μg/mL (n = 3). **n** α-SMA^+^ NHLF cells also show accelerated migration. **o** Type IV collagen-treated cells have a significantly increased migration filled area after 10 hours of removal of insert. Before insert removal, the cells were grown in type IV collagen for 2 days (n = 3). Scale bars = 100 μm in **a**, **b**, **e**, **f**, 200 μm in **c**, **d**, **g**, **h**, **j**–**l**, and 35 μm in **n**

We further characterized the lung mesenchyme in addition to the epithelial and endothelial compartments. The PDGFRα-expressing pulmonary myofibroblast progenitors that normally decorate the walls of terminal saccules at E18.5 (Fig. 6a), showed an irregular patchy distribution in *Col4a1*^*+/Δex41*^ (Fig. 6b), *Col4a1*^*+/G394V*^ and *Col4a2*^*+/G646D*^ (Additional file 7: Figure S7A, B) mutants. *Pdgfrα* mRNA was significantly decreased in *Col4a1*^*+/Δex41*^ lungs (Wilcoxon rank-sum test *P* < 0.05; Fig. 6i). At P6 and P30, the decrease of PDGFRα^+^ cells was evident in *Col4a1*^*+/Δex41*^ (Fig.6c, d and Additional file 8: Figure S8A, B), but not in the conditional mice *R26-Cre*^*ER*^*; Col4a1*^*+/Flex41*^ and in the *Tie2-Cre; Col4a1*^*+/*Flex*41*^ mutants (Additional file 8: Figure S8C, D). Differentiated lung myofibroblasts express specific markers related to their functional state [29]. During saccular formation, α-SMA myofibroblasts, normally detected at the tip of primary septa (Fig. 6e), were decreased in a patchy distribution in *Col4a1* and *Col4a2* mutant lungs (Fig. 6f and Additional file 7: Figure S7E, F). No difference in *α-Sma* gene expression was found in *Col4a1* mutants (Fig. 6i). At P6, α-SMA was not observed at the septal tips (Fig. 6g, h). After alveologenesis, α-SMA^+^ alveolar myofibroblasts were only localized at the tips of alveolar ducts (Fig. 6j) [26]. However, α-SMA^+^ slender interstitial myofibroblasts were present in surviving *Col4a1*^*+/Δex41*^ and in *R26-Cre*^*ER*^*; Col4a1*^*+/Flex41*^ mice (Fig. 6k and Additional file 3: Figure S3G), but not in *Tie2-Cre; Col4a1*^*+/Flex41*^ lungs (Fig. 6l).

Lipofibroblasts are a second type of lung interstitial fibroblast; therefore, we examined lung lipofibroblasts in mutants by staining for adipose differentiation-related protein (ADFP). Lipofibroblasts were found to be moderately increased at P6 and markedly elevated at P30 in the *Col4a1*^*+/Δex41*^ and in *R26-Cre*^*ER*^*; Col4a1*^*+/Flex41*^ compared to normal lungs (Additional file 4: Figure S4B, E, K–M). However, these changes were not observed in *Tie2-Cre; Col4a1*^*+/Flex41*^ mutants (Additional file 4: Figure S4N).

To confirm that the increased lipids in mutants are due to type II pneumocytes and lipofibroblasts, we stained for macrophage antigen (MAC). P6 *Col4a1*^*+/Δex41*^ lungs (Additional file 4: Figure S4C, F) showed a slight decrease of macrophages that was not so evident at P30 (Additional file 4: Figure S4O, R).

Since the data above suggest a role of type IV collagen in alveolar myofibroblast proliferation and mobilization, we investigated myofibroblast proliferation by treating Normal Human Lung Fibroblast (NHLF) with type IV collagen recombinant protein. α-SMA^+^, but not α-SMA^–^, NHLFs showed a statistically significant increase in proliferation and migration after 48 hours of treatment (Fig. 6m–o). These results confirm that type IV collagen is sufficient to induce both myofibroblast proliferation and migration.

### Abnormal expression of tropoelastin and elastin fiber deposition in type IV collagen mutants

**Fig. 7:**
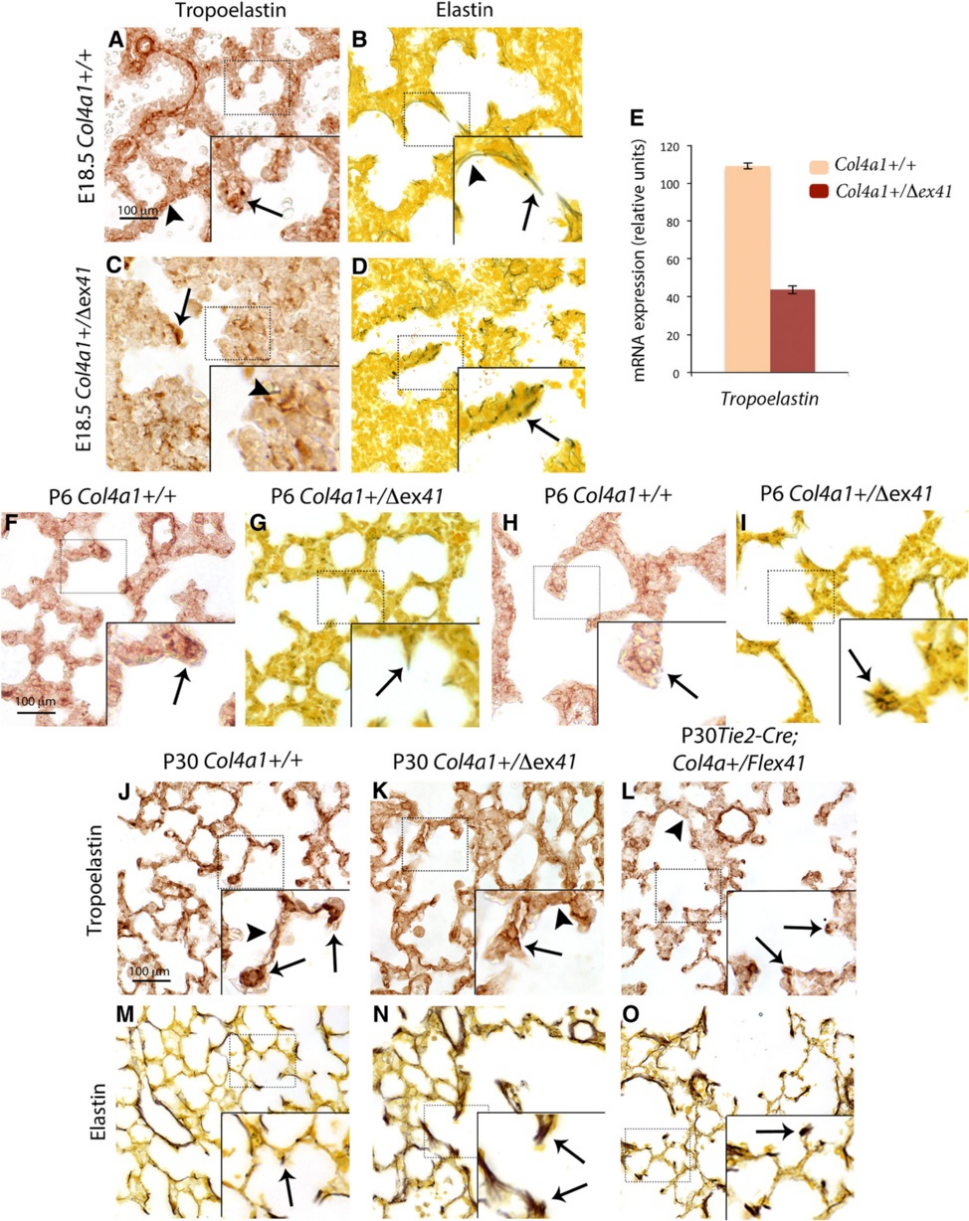
Abnormal alveolar tropoelastin and elastin fiber accumulation. **a** At E18.5, tropoelastin expression in the developing septa in normal lungs is detected at the tips (arrow) and throughout the interstitium (arrowhead). **c** By contrast, in *Col4a1* ^*+/Δex41*^ lungs, tropoelastin expression shows a patchy interstitial accumulation (arrowhead) with abnormal expression in the maldeveloped primary septa (arrow). **b** Hart’s staining marks the lung elastin fibers at E18.5. Control lungs show well-defined thin elastin fibers (black) in the saccule spaces (arrowhead) and at the tip of developing primary septa (arrow). **d** Saccule elastin fibers in *Col4a1* ^*+/Δex41*^ mutants are interstitial and tortuous or fragmented at E18.5 (arrows). **e** Real-time PCR for *Tropoelastin*. The expression of *Tropoelastin* is decreased in *Col4a1* ^*+/Δex41*^ (Wilcoxon rank-sum test *P* < 0.07*). Gapdh* was used as a normalizer. **f**, **g** At P6, tropoelastin is found at the tips of developing septa (arrow) and in elastin fibers (arrow). **h**, **i** Mutants show a decrease of tropoelastin (arrow) and abnormal accumulation of elastin fibers at the septa tips (arrow). **j** At P30, while tropoelastin expression is localized at the tip of secondary septa marking the myofibroblasts (arrow) and in the septal interstitium as a thin line (arrowhead). **k** In *Col4a1* ^*+/Δex41*^ mutants, tropoelastin is localized diffusely throughout the stumpy septal tips and in the interstitium of the secondary septa in a patchy pattern (arrow and arrowhead). **l** *Tie2-Cre; Col4a1* ^*+/Flex41*^ lungs show defined expression of tropoelastin at the tip of the lung septa as do normal lungs, but the septa are reduced in size (arrow). In addition, the less severe *Tie2-Cre; Col4a1* ^*+/Flex41*^ mutants show no tropoelastin localization in the interstitium (arrowhead). **m** After alveolarization, elastin fibers build up on the tip of secondary septa (arrow). **n** In *Col4a1* ^*+/Δex41*^, excessive accumulation of elongated elastin fibers is apparent (arrows). **o** In *Tie2-Cre; Col4a1* ^*+/Flex41*^ elastin fibers are not elongated or ectopic in the interstitium (arrow) when compared to the abnormal elongation apparent in *Col4a1* ^*+/Δex41*^ **n**. Scale bars = 100 μm in **a**–**d** and **f**–**l**

The force necessary for lifting the alveolar crest from the primary septal wall is thought to be produced by septal fibers [25, 49], which prompted us to study tropoelastin and elastin fiber localization in type IV collagen mutants. During saccular formation, the normal distal expression of tropoelastin was reduced in *Col4a1* and *Col4a2* mutants and the elastin fibers were discontinuous and fragmented (Fig. 7a–d and Additional file 7: Figure S7E–H). Real-time PCR showed a borderline significant decrease of tropoelastin in *Col4a1* mutants (Wilcoxon rank-sum test *P* = 0.07; Fig. 7e). At P6, very little expression of tropoelastin was found at alveolar tips and elastin fibers were abnormally deposited (Fig. 7f–i). At the end of alveolarization (P30), tropoelastin and elastin fibers were diffuse throughout the lung interstitium and at the tip of the secondary septa of Col4a1^+/Δex41^ (Fig. 7j, k, m, n) and in *R26-Cre*^*ER*^*; Col4a1*^*+/Flex41*^ (Additional file 3: Figure S3H, I). In *Tie2-Cre; Col4a1*^*+/Flex41*^ mutants, tropoelastin was markedly decreased in the lung interstitium and septal tips (Fig. 7l). Abnormalities in elastin fiber deposition were less severe than in the non-conditional mutants (Fig. 7m, o).

### Type IV collagen directs epithelial–mesenchymal association in vitro

**Fig. 8:**
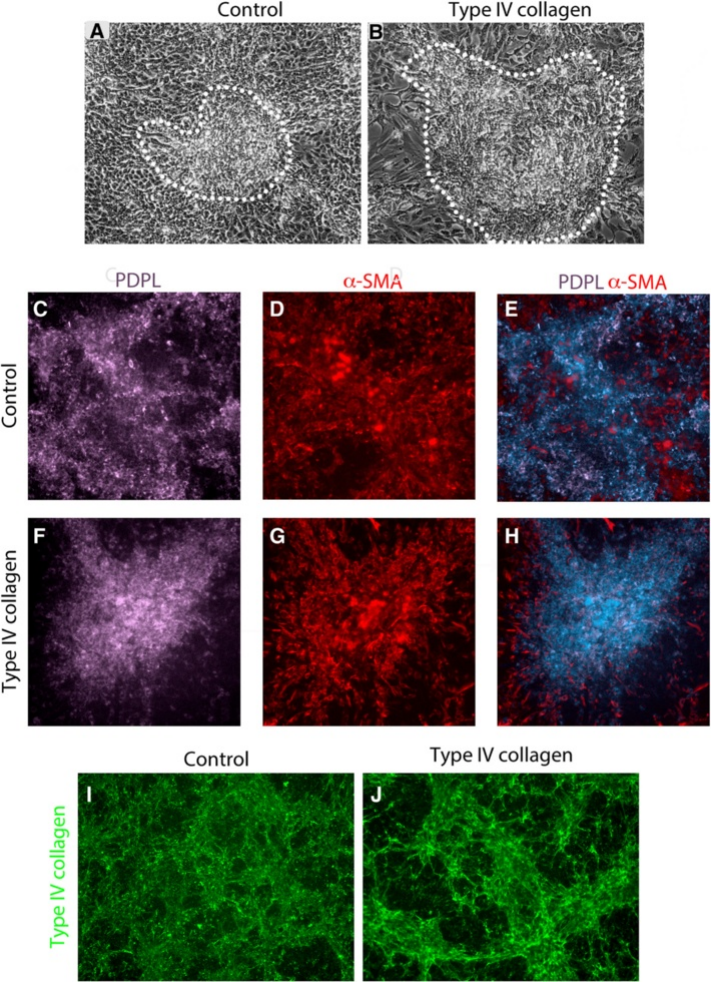
In vitro epithelial–mesenchymal aggregations after *COL4A* treatment. **a**, **b** Control and type IV collagen-treated epithelial–mesenchymal co-cultures. Septum-like formations are outlined. Type IV collagen treatment (5 μg/mL) results in bigger epithelial–mesenchymal aggregations. **c**, **f** Localized PDPL expression is found in controls and, more highly expressed, in type IV collagen-treated co-cultures. **d**, **g** α-SMA staining is dispersed throughout the co-cultures with clear accumulation of α-SMA^+^ of the co-culture aggregations. **e**, **h** Co-localization of PDPL and α-SMA. **i**, **j** Staining of co-culture with COL4A shows increased accumulation of Col4a1-stained condensed fibers

To investigate the role of type IV collagen in septation, we used an epithelial–mesenchymal co-culture model of alveolar morphogenesis [50]. This model produces peaks of mesenchymal cells covered by an epithelial lining. The central core of mesenchymal cells closely resembles the myofibroblasts seen during alveolar septal development in vivo [50]. When MRC-5 fetal mouse mesenchymal cells were co-cultured with human A549 epithelial cells, septal-like formations appeared after 4–5 days and reached their maximum after 7 days (Fig. 8a). After type IV collagen addition, cellular aggregations where larger (Fig. 8b) and showed strong staining of PDPL, α-SMA^+^ and type IV collagen proteins (Fig. 8c–j).

## Discussion

We performed gene expression profiling at four embryonic time points (E14–18) in the chick lung seeking to uncover molecular regulators contributing to late lung development and taking advantage of rapid development of the BGB in the chick. This analysis revealed that *COL4A1* and *COL4A2* genes are highly upregulated during the late embryonic stages of chick lung development. These data are consistent with the peaks of expression of murine Collagen IV at E18 and P7, which correlate with saccular and alveolar development [51]. Our study of *Col4a1* and *Col4a2* mutant mouse lungs revealed multiple abnormalities in pulmonary alveoli supporting the importance of type IV collagen in alveolar development. The phenotype observed in *Col4a1* and *Col4a2* mutants is characterized by a block between the saccular and alveolar stages reminiscent of the severe abnormalities of premature newborns and children with congenital diaphragmatic hernia.

In this work, we show that murine type IV collagen is necessary for alveolar patterning and cellular development during saccular and alveolar morphogenesis. After alveolarization, at P30, all the analyzed mutants showed alveolar simplification with the strongest phenotype in Col4a1^+/Δex41^ lungs. Postnatal-induced conditional mutants in *R26-CreER; Col4a1*^*+/Flex41*^ displayed a milder phenotype than *Col4a1*^*+/Δex41*^ as a result of normal alveolar progenitor development during saccular development. The abnormal phenotype in *R26-CreER; Col4a1*^*+/Flex41*^ revealed an active role of type IV collagen in alveolarization that is independent of branching and saccular defects. We also show that *Col4a1* mutation carried by the Tie2 promoter in *Tie2-Cre; Col4a1*^*+/Flex41*^ was sufficient in causing microvascular defects without the epithelial and fibroblast abnormalities observed in *Col4a1*^*+/Δex41*^ and *R26-CreER; Col4a1*^*+/Flex41*^.

Type IV collagen is found in the basement membranes of epithelial and interstitial endothelial cells where alveolar elastin-producing myofibroblasts are anchored [26, 27]. In addition, normal *Col4a1* mRNA expression is elevated in the lung interstitium and at the tips of the septa in the mouse after branching morphogenesis. Consequently, it is not surprising that pulmonary epithelial, vascular and myofibroblast development is affected by *Col4a1* or *Col4a2* mutations. At the completion of branching morphogenesis (E16.5), we did not observe differences between *Col4a1*^*+/Δex41*^ and *Col4a1*^*+/+*^ lungs. The absence of branching may be because we analyzed heterozygous mutants in which sufficient levels of native protein may be enough to carry out branching. Previous studies have also shown that defects on basement membrane components affect alveoli formation, but not branching morphogenesis [43, 44]. In addition, our data using the postnatal conditional mutants suggests a role of Col4a in alveologenesis independent of branching.

By the time of saccular (E18.5) morphogenesis, the lungs from mutant mice had a thick interstitium and failed to develop normal saccules. We showed that mutant lungs have a decrease in the number of distal SOX9^+^ epithelial progenitors and an increased number of type II pneumocytes in a patchy distribution. This data suggests that type IV collagen may regulate *Sox9*, which has been previously shown to be critical for alveologenesis by controlling the transition of distal epithelium from proliferation to differentiation [52]. In mutants, a decrease in *Sox9* is coincident with expansion of type II pneumocytes. Defects in epithelial differentiation may also be a consequence of abnormal basement membrane, which normally undergoes alteration during lung development [53–55]. Starting in the saccule stage and during alveolarization the basement membrane is discontinuous under type II pneumocytes but not under type I [53, 54]. Therefore, it is also possible that the mutant type IV collagen may affect basement membrane integrity, altering type II pneumocyte differentiation. Epithelial differentiation defects in mutants are also supported by the decrease in HOPX cells. Epithelial progenitors express both type I and II pneumocyte markers [3]. Hence, the increase of pSPC^+^ cells appears to be a consequence of differentiation but not of alveolar progenitor proliferation. Increased and disorganized type II pneumocytes and decreased HOPX were also observed in postnatal *Col4a1*^*+/Δex41*^ and induced *R26-Cre*^*ER*^*; Col4a1*^*+/Flex41*^ mice, but not in vascular directed *Tie2-Cre; Col4a1*^*+/Flex41*^ conditional mutants. These findings indicate that type IV collagen controls epithelial differentiation and organization independently of vascular type IV collagen.

It has been shown that mutations in *Col4a1* can affect the integrity of the vascular basement membrane leading to an aberrant organization of capillary structures [10, 15, 17, 56]. Additionally, mice with mutations in the basement membrane component *Fibulin* have abnormally wide lung capillaries with normal capillary organization [42]. Our *Col4a1* and *Col4a2* mutant mice likewise manifested aberrant organization of the pulmonary microvasculature that was obvious 1 month after birth due to an apparent abnormal angiogenesis. Pulmonary endothelial growth is known to be essential for alveolar formation not only in lung embryonic development [21, 37, 57–59], but also in pulmonary alveolar regeneration [60]. Our work shows that *Col4a1* mutation in vascular endothelium is sufficient to produce abnormalities in pulmonary microvascular growth, to alter the alveolar endothelial–epithelial association, and to cause alveolar epithelial developmental deficiencies. More experiments are needed to address, conversely, whether restricted type IV collagen mutation in the epithelium will also direct abnormalities in the association of the endothelium and epithelium of the alveolar interface.

During alveologenesis the pulmonary saccules are divided through serial septation, which increases the surface area for gas exchange. Important players in the process of septation are myofibroblasts anchored in the alveolar basement membrane [26–28]. The expression of *PDGFRα* is decreased in *Col4a1* mutants and both PDGFRα^+^ and α-SMA^+^ alveolar myofibroblasts are displaced in the interstitium of the embryonic *Col4a1* and *Col4a2* mutants. The decrease of PDGFRα^+^ and α-SMA^+^ cells is also evident after birth. This suggests that type IV collagen may be necessary for the correct spatial patterning of the alveolar myofibroblasts during septal formation in part through the PDGFRα pathway. In Pdgfrα^–/–^ mice the alveolar myofibroblast spreading is compromised and most PDGFRα^+^ cells remain in clusters at the bronchiolar wall instead of being scattered throughout the alveolar sacs [31]. In contrast to myofibroblasts, *Col4a1* mutants have an increase in lipofibroblasts. Recently, it has been reported that the PDGFRα^+^ fibroblast lineage contributes to the lipofibroblast pool in the mouse lung [61] and that myofibroblasts and lipofibroblasts are characterized by PDGFRαGFP^bright/high^ and PDGFRαGFP^dim/low^ expression during alveolarization [62, 63].

In the presence of type IV collagen mutant protein, both myofibroblast proliferation and migration were negatively affected, while in vitro addition of type IV collagen protein induced proliferation and migration in α-SMA^+^ lung fibroblasts, confirming its important role.

Alveolar myofibroblasts are the source of alveolar tropoelastin, which is an important regulator of alveologenesis when its expression levels are high [51]. α-SMA expression in elastogenic alveolar myofibroblast cells follows type IV collagen expression, and both precede the expression of tropoelastin [51]. Interestingly, *Col4a1* mRNA is expressed in the interstitium and at the tips of alveolar septa in a pattern similar to tropoelastin. Therefore, it is conceivable that type IV collagen regulates elastogenesis specifically in the α-SMA^+^ cell population. Earlier loss of type IV collagen directs loss of alveolar myofibroblasts, tropoelastin, and causes aberrant elastin fiber accumulation which may be the main cause for the dramatic failure of saccular formation. By the end of alveolarization in the mouse, at P30, lungs appear to have excessive interstitial elastin fiber accumulation in *Col4a1*^*+/Δex41*^ and *R26-Cre*^*ER*^*; Col4a1*^*+/Flex41*^ mice which is likely due to ectopic elastin-producing α-SMA^+^ myofibroblasts localized in a patchy distribution in the lung interstitium. It is also possible that the abnormal elastin fiber accumulation may be a compensatory response to failure of alveolar formation [32]. *Tie2-Cre; Col4a1*^*+/Flex41*^ lungs lack this patchy interstitial distribution of α-SMA^+^ cells and interstitial elastin fiber accumulation, suggesting a direct effect of *Col4a1* on alveolar myofibroblasts that is independent of vascular type IV collagen*.*

There are four alpha integrins (ITGA) known to bind to collagen type IV – ITGA1, ITGA2, ITGA10, ITGA11. Among them, ITGA1 and ITGA2 have a higher affinity for type IV collagen than ITGA2 and ITGA11 [64, 65]. ITGA2 is expressed in proximal and distal lung epithelium co-localizing with pSPC (Additional file 9: Figure S9A, B), and ITGA1 is expressed in the microvasculature by co-staining with CD31. ITGA2 is also expressed in non-type II cells, suggesting a role in other developmental processes in the lung. ITGA11 has been reported to be expressed in human lung interstitial fibroblasts [66], but we only observed it in the proximal epithelium (data not shown). Future investigation should be directed at uncovering the molecular pathways by which integrins affect type IV collagen regulation of the epithelium, vasculature and myofibroblasts during alveologenesis.

To address a direct effect of type IV collagen in septal formation we used an in vitro co-culture of mesenchymal–epithelial cells designed to resemble alveolar septal formation [50]. A549 epithelial cells share properties with immature alveolar type II pneumocytes but not of type I pneumocytes (data not shown and [67, 68]). When A549 cells are co-cultured with lung fibroblasts, they express the type I pneumocyte marker PDPL, which co-localizes with α-SMA^+^ cells in type IV collagen treated co-cultures. Endogenously produced type IV collagen fibers are also increased in the cell aggregates found in treated co-cultures. These aggregates may be due to cellular reorganization in addition to epithelial–mesenchymal proliferation. Herein, we demonstrated that type IV collagen signaling might indeed be important for the epithelial–mesenchymal interaction needed for septal formation.

**Fig. 9:**
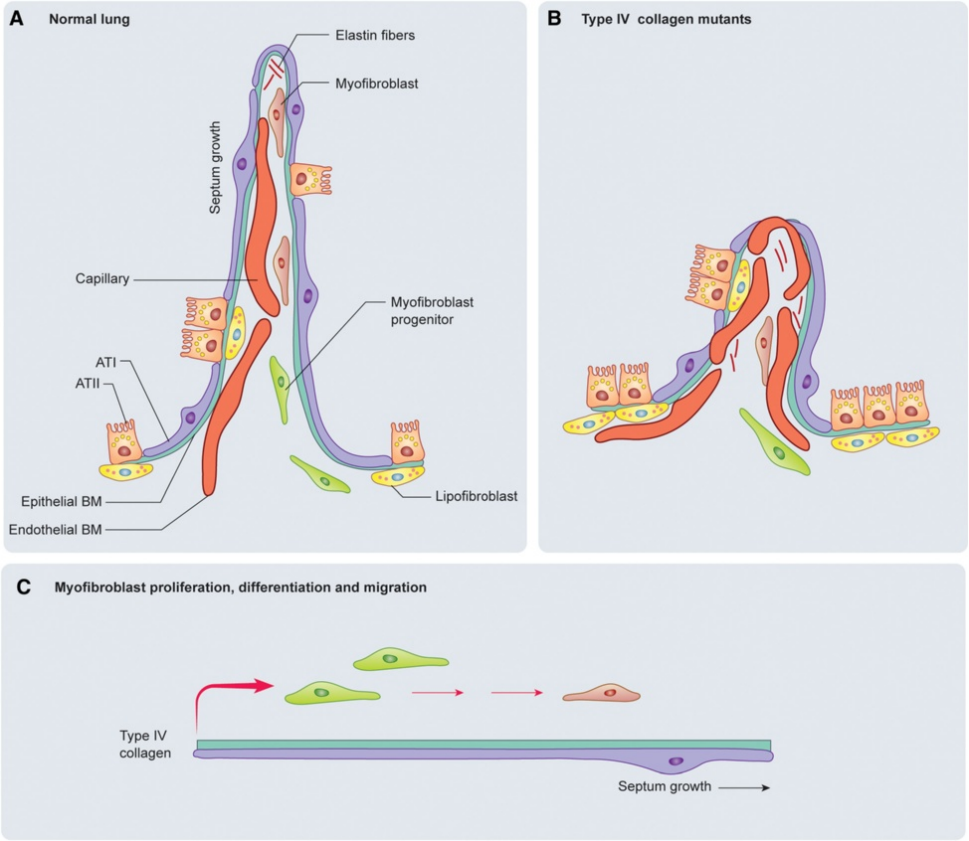
Schematic alveolar representation and suggested model for type IV collagen during alveologenesis. **a** Normal alveolar septum in which type I (ATI) and II pneumocytes (ATII) cover the alveolar surface with the blood capillaries in close association. Alveolar myofibroblast progenitors localized at the base of the septum proliferate, differentiate into alveolar myofibroblast, and migrate where they synthesize elastin. Lipofibroblasts are localized close to ATII. **b** Type IV collagen mutations cause abnormal septum formation. Type IV collagen lungs have increased cells with lipid content (ATII and lipofibroblasts), and abnormal capillary formation. Alveolar myofibroblasts fail to proliferate, differentiate and migrate, causing defects in elastin deposition. **c** Suggested model for type IV collagen, basement membrane and septal myofibroblast development. Type IV collagen is a central player in myofibroblast progenitor proliferation, differentiation and mobilization to the tip of the septum

In summary, we show that type IV collagen is essential for alveolar lung patterning and propose a model (Fig. 9a, b) in which type IV collagen regulates the epithelial and endothelial components important for alveologenesis (Fig. 9c), induces interstitial α-SMA myofibroblasts to proliferate and migrate to the tip of the septa during early septation, and later contributes to extension and final maturation of secondary septa.

## Conclusion

We conclude that type IV collagen is a key player in the process of alveolar morphogenesis and is critical for the proper formation of the BGB and the process of septation. *COL4A1* and *COL4A2* mutations in humans cause multisystem disorders in which pulmonary symptoms have not been observed. However, Goodpasture syndrome, an autoimmune disease in which antibodies attack the basement membrane *COL4A3* of lungs and kidneys, manifests lung abnormalities ranging from symptoms as mild as a dry cough and minor breathlessness to those with more severe lung damage [11]. It is possible that mutations in *COL4A1* and *COL4A2* affecting the lung could go undetected, as they would cause major developmental abnormalities leading to early mortality.

## Methods

### Normal Chick embryos

Timed fertilized white leghorn eggs (SPAFAS, CT, USA) were incubated in a humidified incubator (Khul, NJ) at 38 °C and staged by embryonic day (E) and managed as previously described [69].

### RNA isolation and double stranded cDNA synthesis

Total RNA was isolated from embryonic chick lungs harvested at embryonic days E14, E15, E16 (for each time point n = 3), and E18 or E18.5 from *Col4a1*^*+/Δex41*^ mice lungs (Controls n = 10 and *Col4a1*^*+/Δex41*^ = 10). RNeasy Mini Kit (Qiagen, Valencia, CA, USA) was used to isolate total RNA according to the manufacturer’s instructions. Double stranded cDNA was synthesized from total chick lungs RNA using a SuperScript Double-Stranded cDNA Synthesis Kit (Life Technologies; Grand Island, NY, USA) following the manufacturer’s instructions. All cDNA samples were tested for purity and integrity at Roche Applied Science (Indianapolis, IN, USA) using a cDNA LabChip on an Agilent 2100 Bioanalyzer prior to labeling.

### Microarray processing and analysis

Chick samples were labeled by Roche Applied Science as previously described [70]. After labeling, the samples (n = 3 for each time point) were hybridized for gene expression analysis to a *Gallus Gallus* custom 12 × 135 K microarray (NimbleGen Eukaryotic Gene Expression HD2 12-Plex Array Delivery) which covers 22,000 open reading frames. Microarray raw data was normalized by Robust Multi-array Analysis and analyzed using DNASTAR ArrayStar software package for analysis. One-way ANOVA was performed at a 95 % confidence limit. The data discussed in this publication have been deposited in NCBI’s Gene Expression Omnibus [71] and are accessible through GEO Series accession number GS72385 (https://www.ncbi.nlm.nih.gov/geo/query/acc.cgi?acc=GSE72385).

### Ontology assessment

The *Gallus gallus* transcript IDs were converted to their murine homologous and named by Ensembl with BioMart (http://useast.ensembl.org/index.html). Once the murine homologous gene list was generated, functional classification of genes was performed using MGI and Vlad (http://proto.informatics.jax.org/prototypes/vlad/).

### Mouse type IV collagen models

Procedures were performed in accordance with Institutional Animal Care and Use Committee guidelines IACUC, protocol #AN102193-02B. *Col4a1*^*+/Δex41*^ (E16.5 n = 12; E18.5 n = 4; P6 n = 2; P30 n = 5), *Col4a1*^*+/G394V*^ (n = 3), and *Col4a2*^*+/G646D*^ (n = 5) mutant mice were described previously [45, 56]. To examine the role of type IV collagen in alveologenesis, we used the inducible conditional *R26-Cre*^*ER*^*; Col4a1*^*+/*Flex41^ mice in which deletion of exon 41 of *Col4a1* is induced by postnatal injection of tamoxifen (which activates the CRE recombinase) in the pups at birth [16]. Pups were injected with 50 μg of tamoxifen intragastrically (10 mg/mL; Sigma-Aldrich, St Louis, MO) for three consecutive days. The vascular role of *Col4a1* in alveolar development was examined by the previously described vascular driven *Tie2-Cre; Col4a1*^*+/Flex41*^ mice [16]. Lungs from *Tie2-Cre; Col4a1*^*+/Flex41*^ (n = 5) or *R26-Cre*^*ER*^*; Col4a1*^*+/Flex41*^ (n = 5) mice were harvested 30 days after birth (P30) for analysis. All mutant mice analyzed were heterozygous.

### Quantitative PCR

The RNA extracted previously for conducting microarrays from E14, E15, E16, and E18 (n = 3) chick lungs or *Col4a1*^*+/Δex41*^ lungs from E18.5 normal (n = 11) and *Col4a1*^*+/Δex41*^ (n = 10) mouse embryos were reverse-transcribed to cDNA using superscript (Life Technologies, Grand Island, NY, USA) following the manufacturer’s protocol for RT-qPCR, which was carried out in triplicate for each sample with IQ SYBR Green Supermix (Biorad, Hercules, CA, USA) and gene-specific primers. The data was analyzed using the ΔΔC_t_ method on the BioRad CFX manager v1.5. The most stable housekeeping genes were selected by geNorm as normalizers for the Real-Time PCR experiments: chick G6PDH: 5′CGGGAACCAAATGCACTTCGT3′, 5′CGCTGCCGTAGAGGTATGGGA3′ and mouse *Gapdh:* 5′GGGATGCAGATCTTCGTGAAA3′; 5′CTTGCAGCAAAGATCAACCT3′. Chicken primers: COL4A1: 5′TGAAAGAGCACACGGTCAAG3′, 5′ATGGGTTCAGGAGTGGACAG3′; and COL4a2: 5′CCGGGTCGCAGCGTTAGCAT3′, 5′AGCCAGCCAGCCCTAGGTCC3′. Mouse primers; *Cd31* 5′GAAGTGTCCTCCCTTGAGCC′, 5′GGAGCCTTCCGTTCTTAGGG3′; *Tropoelastin:* 5′CAGTTCCACTC3′, 5′GATTCGGCGTC3′; *Nkx2.1*: 5′CAGTTCCACTCTGCAACGGA3′, 5′CGATTCGGCGTCGGCTGG3′; *Pdgfr-α*: 5′CCGGGTCGCAGCGTTAGCAT3′, 5′AGCCAGCCAGCCCTAGGTCC3′; *pSpc:* 5′TATGACTACCAGCGGCTCCT3′, 5′CCAGCTTAGAGGTGGGTGTG3′; *α-Sma:* 5′CCGGGTCGCAGCGTTAGCAT3′, 5′AGCCAGCCAGCCCTAGGTCC3′; *Sox9:* 5′AGGAAGTCGGTGAAGAACGG3′, 5′GGACCCTGAGATTGCCCAGAA3′. RT-qPCR mRNA expression was analyzed by the Wilcoxon rank-sum under the hypothesis of H1 = a < b. We reasoned H1 as RT-qPCR assay followed protein cell counting in which we already predicted that a < b (supporting data are available in Additional file 10).

### Tissue processing

Mouse embryos were fixed with 4 % paraformaldehyde in RNAse-free PBS overnight at 4 °C. Fixed embryos and harvested lungs were washed in PBS with 0.1 % Tween 20 (PBT) and either taken through a graded series of methanol/PBT washes or stored at –20 °C in 100 % methanol. General cytoarchitecture was primarily examined using H&E staining, on 5-μm paraffin sections prepared by standard protocols [72]. For semi-thin sections, lung samples were fixed in 4 % buffered glutaraldehyde followed by dehydration in graded ethanol, and the samples were embedded in a Polybed/Araldite 6500 mixture (Polysciences, Warrington, Pennsylvania, USA). The 1-μm thick semi-thin sections were stained with toluidine blue. The ultrathin sections were contrasted with uranyl acetate and lead citrate and studied with an H-7600 Hitachi electron microscope.

### Immunohistochemistry

Immunohistochemical staining was performed using standard techniques. Antigen retrieval was achieved by heat treatment in a microwave oven for 20 min at low power in 0.01 M sodium citrate buffer at pH 6. Before antibody incubation, peroxidase was quenched with H_2_O_2_. Biotinylated secondary antibody (Vector Laboratories Inc.) was used to localize antibody antigen complexes in the ABComplex/HRP detection system (Dako, Carpinteria, CA, USA) following the manufacturer′s directions. Antigen detection was enhanced with 3,3’-diaminobenzidine (Vector Laboratories Inc.). In tissue detected by fluorescence, secondary Alexa Fluor antibodies were used (Life Technology, Grand island, NY, USA). The following antibodies were used (full list of antibodies in the study): anti-α-SMA (1:200; rabbit polyclonal; Abcam, Cambridge, MA, USA), anti-prosurfactant protein C (1:400; rabbit polyclonal; Abcam), anti-Ki67 (1:200; rabbit polyclonal; Abcam), anti-mouse Ki67 (1:200; rat monoclonal; Affymetrix eBioscience, San Diego, CA, USA), anti-CD31 (1:25; rabbit polyclonal; Abcam), anti-PDGFR-α (1:50; rabbit polyclonal; SantaCruz, Dallas, TX, USA), anti-PDGFR alpha (1:50; rabbit polyclonal; ThermoFisher, Cambridge, MA, USA), anti-podoplanin (hamster monoclonal; SantaCruz), anti-TTF1 (mouse monoclonal; SantaCruz), anti-SOX9 (rabbit polyclonal; a generous gift from Dr. de Santa Barbara, University of Montpellier, France), anti-tropoelastin (1:150; rabbit polyclonal; a generous gift from Dr. Mecham, Washington University, St. Louis, USA), anti-HOP (1:100; rabbit polyclonal; SantaCruz); anti-E-Cadherin (1:100; mouse monoclonal; Abcam), anti-macrophage MAC (1:100; mouse monoclonal; Abcam), anti-ADFP (1:500; rabbit polyclonal; Novus Biologicals, Littleton, CO, USA), anti-integrin α2 (1:50; mouse monoclonal; SantaCruz), anti-human integrin α1 (1:50; goat polyclonal; R&D Systems, Minneapolis, MN, USA). Images were obtained using a Nikon Eclipse 80i microscope at 20, 40, or 100× magnification, and Spot Imaging software. Blinded epithelial cell counting was performed by two different individuals and analyzed with the ImageJ software. Epithelial counting was examined in a combination of *Col4a1*^*+/Δex41*^, *Col4a1*^*+/G394V*^ and *Col4a2*^*+/G646D*^ (n = 3) and control (n = 3) in technical triplicated and by two-tailed Student’s *t* test (supporting data are available in Additional file 10).

### In situ hybridization

A 561-bp segment of the *Col4a1* transcript (uc009kvb.2) was PCR amplified (PCR Master Mix, Promega, Madison, WI) with one set of exon-exon boundary overlapping primers, designed using Primer-BLAST [73], hosted in the National Center for Biotechnology Information (NCBI) (http://www.ncbi.nlm.nih.gov/). The purified PCR fragment was cloned into the pCR™II-TOPO® TA vector (TOPO® TA Cloning® Kit, Dual Promoter) (ThermoFisher, Life Technologies Corporation), and transformed into One Shot® TOP10 Chemically Competent Cells (ThermoFisher, Life Technologies Corporation). Transformed colonies on agar plates were selected by ampicillin resistance after one night at 37 °C. Sanger sequencing was used to determine the orientation of the insert after linearization of the vector with SpeI restriction digestion (New England Biolabs, Inc.). Sense and anti-sense Digoxigenin-11-UTP labeled probes (DIG RNA Labeling Mix, Sigma-Aldrich) were synthesized with SP6 and T7 RNA polymerases, respectively. In situ hybridization was performed using a technique minimally altered from previously published protocols [74] and developed using the BM purple AP substrate (Roche, Indianapolis, USA) as per the manufacturer’s instructions.

### Hart’s staining

Mice lung paraffin samples were dewaxed and incubated with Hart’s stain for 1 h. After the samples were washed with running tap water for 10 min and rinsed with distilled water, they were counterstained with Van Gieson’s Solution for 1 min. Samples were dehydrated in 95 % alcohol, cleared in Xylene, and mounted for analysis.

### Measurement of cell growth by methyl thiazol tetrazolium (MTT) assay

Cell proliferation and viability assays were evaluated by MTT assay; 96-well plates were coated with 0, 1 or 5 μg/mL human type IV collagen recombinant protein for 2 hours at room temperature (Millipore; Darmstadt, Germany). Following the manufacturer’s directions (Lonza Group Ltd, Basel, Switzerland), NHLFs were cultured at a density of 1000 cells/well in 100 μL growth medium and incubated at 37 °C, 5 % CO_2_ for 1, 3 and 5 days, when culture medium was aspirated and 10 % MTT [7.5 mg/mL 1× PBS] was added to the existing 100 μL growth FGM-2 media supplemented with FBS (Lonza, MD, USA). After incubation for 2 hours at 37 °C, the dissolving solution (70 % isopropanol, 0.028 % Triton-X 100, 0.0028 % HCL) was added for 15 min at room temperature. Absorbance at 595 nm was measured using a microplate reader (Molecular Devices, Sunnyvale, CA, USA). Experiments were conducted in technical triplicate and analyzed by two-tailed Student’s *t* test (supporting data are available in Additional file 10).

### Migration assay

Between 5000 and 6000 NHLF cells expressing α-SMA were plated in 12-well plates with silicone culture inserts (Ibidi, GmbH, Planegg, Germany) for 48 hours with either 5 μg/mL type IV collagen protein or 0.5 M acetic acid as a control. Inserts were removed, fresh treatment added, and cell migration monitored during 24 hours. Images of the different time points captured on a Nikon DS-QiMc camera using NIS-Elements BR 3.0. Quantification of cell invasion into the insert space was performed and compared using ImageJ software. Experiments were conducted three times (n = 3) in triplicate. Gap cell invasion was analyzed by two-tailed Student’s *t* test.

### Epithelial–mesenchymal co-culture

Human embryonic lung fibroblast MRC-5 cells (ATCC, VA, USA) and human lung carcinoma epithelial A549 cells (ATCC) were cultured in either DMEM or EMEM with 10 % FBS at 37 °C and 5 % CO_2._ Co-culture experiments followed published protocols [50] with minor modifications. Briefly, MRC-5 fibroblast cells were plated at high density and grown to confluence in EMEM for 5–6 days. A549 epithelial cells were added to MRC-5 cells at high density (250,000 cells per well in a 12-well plate) to ensure full coverage over the underlying MRC-5 cells. Co-culture was maintained in DMEM with 10 % FBS and changed to 2 % after plaiting the A549 cells.

Type IV collagen protein (5 μg/mL; EMD Millipore, Billerica, MA, USA) was added in solution after overnight attachment of A549 cells. Type IV collagen was added fresh every 2 days. After 5 days of treatment, samples were fixed, immunolabeled for alveolar markers and mounted for imaging using standard techniques. Nuclei were labeled with DAPI (Vectashield; Vector Laboratories Inc., Burlingame, CA, USA). Images were obtained using Nikon Eclipse TS100 at 4× magnification using NIS-Elements BR 3.0. Experiments were conducted in triplicate.

## Additional files

Additional file 1: Figure S1. Heatmap and volcano plot of microarray data. (A) Heatmap of differentially expressed genes across the four time points. Samples cluster according to time point (blue: E18, green: E16, red: E14 and yellow: E15). The unsupervised hierarchical clustering of all the transcripts forms six discrete groups. From top to bottom, cluster A (n = 190 chick transcripts) contains genes involved in neurogenesis, neuron differentiation and neuron projection morphogenesis; cluster B (n = 206) is enriched for hemostasis, coagulation and immune system process genes; cluster C (n = 42) contains various genes involved in miscellaneous processes; cluster D (n = 119) is characterized by angiogenesis, vascular development, endothelial and smooth muscle proliferation genes; cluster E (n = 84) groups genes, mostly transcription factors, with a role in organogenesis. Finally, cluster F (n = 18, indicated by the red asterisk) contains a limited number of genes involved in blood vessel morphogenesis and includes *Adam12*, *Col4a1*, *Col4a2*, *Crmp1*, *Emp1*, *Epas1*, *Fbln5*, *Meis1*, *Mgp*, *Podxl*, *Snai2*, and *Wnt11*. GO enrichment analyses were computed by the Panther Classification System, available at http://geneontology.org. GO annotations were retrieved after one-to-one conversion of chick to mouse orthologs by Ensembl Biomart, accessible at http://www.ensembl.org/biomart/martview. (B) A volcano plot was used to compare fold change from E14 to E18 of log2 normalized data (x-axis) and –log10 of the adjusted *P* value across the four time points, computed by limma. Transcripts with adjusted *P* values less than or equal to 0.05 and fold change greater than, or equal to, 1 are shown in green. The *Col4a1* and *Col4a2* transcripts are indicated by the red and blue squares (top right), respectively. (C) Bi-plot of the Principal Component Analysis results, conducted using the prcomp function to identify potential sample outliers. (TIF 16074 kb) Additional file 2: Figure S2. Histology and immunohistochemistry analyses of the distal epithelium of *Col4a1* ^*+/G394V*^ and *Col4a2* ^*+/G646D*^ mutants. (A, B) Hematoxylin and eosin staining shows thickened interstitium and the small primitive alveolar sacs in *Col4a1* and *Col4a2* mutant lungs. Immunohistochemistry of NKX2.1 (C, D), SOX9 (E, F) and PSPC (F, G) in *Col4a1* ^*+/G394V*^ and *Col4a2* ^*+/G646D*^ display clusters of the distal epithelium. Scale bars = 200 μm in A to G. (TIF 24701 kb) Additional file 3: Figure S3. (A–D) Histology of *R26-Cre* ^*ER*^ *; Col4a1* ^*+/Flex41*^ and *Tie2-Cre; Col4a1* ^*+/Flex41*^ mutants, and (E–I) immunohistochemistry analysis of PDPL-pSPC, HOPX, α-SMA, tropoelastin, and elastin in *R26-Cre* ^*ER*^ *; Col4a1* ^*+/Flex41*^. (A, B) Hematoxylin and eosin shows that both *R26-Cre* ^*ER*^ *; Col4a1* ^*+/Flex41*^ and *Tie2-Cre; Col4a1* ^*+/Flex41*^ mutants have simplified alveolarization. (C) *R26-Cre* ^*ER*^ *; Col4a1* ^*+/Flex41*^ septa are thick and with numerous blood capillaries (arrowheads) and cells with lipid content (arrows). (D) *Tie2-Cre; Col4a1* ^*+/Flex41*^ septa are small and short with increases in blood capillaries (arrowheads), but not in cells with lipid content. (E) pSPC and PDPL co-staining shows a disorganized alveolar epithelium. (F) *R26-Cre* ^*ER*^ *; Col4a1* ^*+/Flex41*^ display a decrease of type I pneumocytes as shown by nuclear staining of HOPX. (G–I) Abnormal localization of α-SMA, tropoelastin and elastin in the septa of *R26-Cre* ^*ER*^ *; Col4a1* ^*+/Flex41*^ lungs*.* Scale bars = 200 μm in A and B, 35 μm in C and D, 50 μm in E, and 100 μm in F to I. (TIF 24706 kb) Additional file 4: Figure S4. Immunofluorescence of pSPC, ADFP and MAC in *Col4a1* ^*+/∆ex41*^, *R26-Cre* ^*ER*^ *; Col4a1* ^*+/Flex41*^, and *Tie2-Cre; Col4a1* ^*+/Flex41*^. (A, D, G–J) pSPC normal localization at P6 (A) and P30 (G) is spread throughout the lung alveoli, while in P6 and P30 *Col4a1* ^*+/Flex41*^ (D, H) and in P30 *R26-Cre* ^*ER*^ *; Col4a1* ^*+/Flex41*^ (I) it is patchy and a slightly increased. This patchy *Col4a1* ^*+/∆ex41*^ and *R26-Cre* ^*ER*^ *; Col4a1* ^*+/Flex41*^ distribution of pSPC is not observed in *Tie2-Cre; Col4a1* ^*+/Flex41*^ (J). (E, L, M) Lipofibroblast staining with ADFP marker shows increased of ADFP^+^ cells in P6 and P30 *Col4a1* ^*+/∆ex41*^ (E, L) and in P30 *R26-Cre* ^*ER*^ *; Col4a1* ^*+/Flex41*^ (M) lungs compared with normal lungs (B, K). (N) *Tie2-Cre; Col4a1* ^*+/Flex41*^ displays decreased ADFP^+^ cells. (C, F) Decrease of MAC^+^ cells in *Col4a1* ^*+/∆ex41*^ (F) at P6, but not as clear at P30 (O–R). Scale bars = 200 μm in A to J and O to R, 100 μm in K to N. (TIF 24708 kb) Additional file 5: Figure S5. (A–F) Immunohistochemistry analyses of E16.5 *Col4a1* ^*+/Δex41*^. (A, B) Double immunofluorescence Ki67 and NKX2.1 in *Col4a1* ^*+/Δex41*^ and wild type lungs show no differences in overall proliferation. Mutants display no differences in SOX9 (C, D), pSPC and NKX2.1 (E, F), CD31 and NKX2.1 (G, H), α-SMA and E-CAD (I, J), or HOPX (K, L) localization. Scale bars = 100 μm in A and B, 200 μm in C to L. (TIF 24679 kb) Additional file 6: Figure S6. Immunofluorescence of PDPL and CD31 *Col4a1* ^*+/G394V*^, *Col4a2* ^*+/G646D*^ mutants, and *R26-Cre* ^*ER*^ *; Col4a1* ^*+/Flex41*^. (A, B) PDPL and CD31 staining shows vascular disorganization around collapse saccules in *Col4a1* ^*+/G394V*^ (A) and *Col4a2* ^*+/G646D*^ (B). This disorganization is also observed in P30 *R26-Cre* ^*ER*^ *; Col4a1* ^*+/Flex41*^ (C, D). Scale bars = 100 μm in A and B, 200 μm in C. (TIF 24703 kb) Additional file 7: Figure S7. Immunohistochemistry of PDGFRα, α-SMA, tropoelastin, and elastin in *Col4a1* ^*+/G394V*^ and *Col4a2* ^*+/G646D*^ lungs. *Col4a1* and *Col4a2* mutant lungs exhibit a decreased and patchy distribution of PDGFRα (A, B), SMA-α (C, D), and tropoelastin expression (E, F). Elastin fibers are atypical and decreased in *Col4a1* ^*+/G394V*^ (G) and *Col4a2* ^*+/G646D*^ (H) lungs. Scale bars = 100 μm in A to H. (TIF 24703 kb) Additional file 8: Figure S8. PDGFRα localization in P30 mutant lungs. (A–D) At P30, *Col4a1* ^*+/Δex41*^ (B) has fewer PDGFRα^+^ cells compared to normal (A), *R26-Cre* ^*ER*^ *; Col4a1* ^*+/Flex41*^ (C) and *Tie2-Cre; Col4a1* ^*+/Flex41*^ (D) lungs. Scale bars = 100 μm in A to D. (TIF 24700 kb) Additional file 9: Figure S9. (A, C) ITGA2 and ITGA1 localization in normal lungs. (A–C) During development, at E18.5, ITGA2 displays epithelial localization that partially co-localizes with pSPC (A, B). At P30, ITGA1 expression is co-stained with CD31, marking the vasculature (C). Scale bars = 100 μm in A–C. (TIF 24700 kb) Additional file 10. Supporting data. (XLSX 14 kb)
