# Erratum to: Type IV collagen drives alveolar epithelial–endothelial association and the morphogenetic movements of septation

**DOI:** 10.1186/s12915-016-0297-7

**Published:** 2016-09-01

**Authors:** Maria Loscertales, Fotini Nicolaou, Marion Jeanne, Mauro Longoni, Douglas B. Gould, Yunwei Sun, Faouzi I. Maalouf, Nandor Nagy, Patricia K. Donahoe

**Affiliations:** 1 The Pediatric Surgical Research Laboratories, Massachusetts General Hospital, Boston, MA 02114 USA; 2 Department of Surgery, Harvard Medical School, Boston, MA 02115 USA; 3 Departments of Ophthalmology and Anatomy, Institute for Human Genetics, University of California, San Francisco, School of Medicine, San Francisco, CA 94143 USA; 4 Department of Human Anatomy, Histology and Embryology, Faculty of Medicine, Semmelweis University, Budapest, 1094 Hungary; 5 Broad Institute of MIT and Harvard, Cambridge, MA 02142 USA

## Erratum

Unfortunately, the original version of this article [1] contained errors. Figure 1 was included incorrectly. The corrected Fig. 1 has been corrected in the original article and Fig. 1 is included correctly below.

**Fig. 1:**
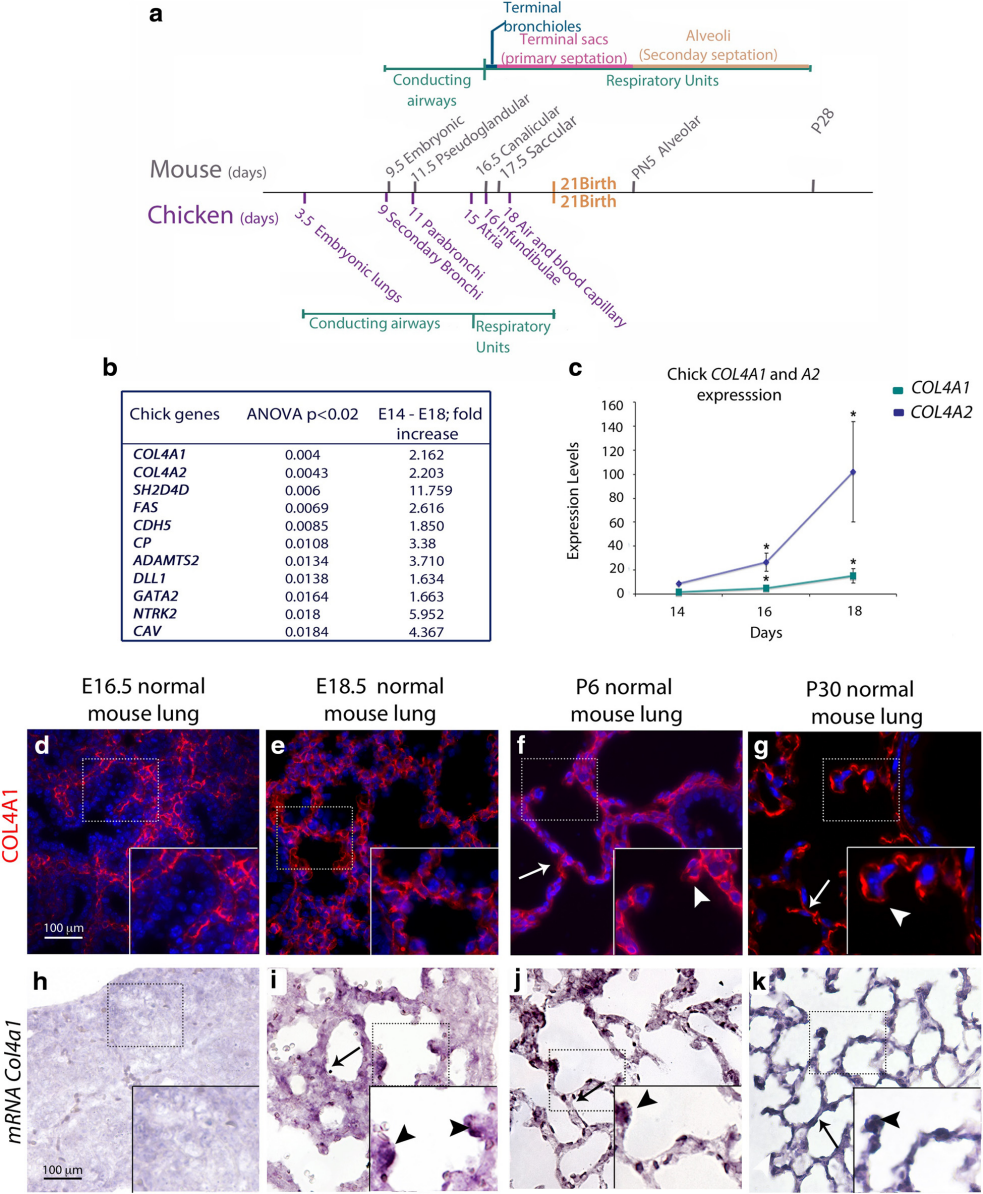
Lung development timeline and type IV collagen expression in the chicken and the mouse. **a** Mouse and chicken lung development comparative timeline. **b** Microarray analysis shows that vascular related genes, among which are *Col4a1* and *Col4a2*, show the highest significance in late chick lung development. **c** Real-time PCR shows differential expression of *Col4a1* (blue) and *Col4a2* (green) between E16 and E18 in chick lungs. *Col4a1* and *Col4a2* expression increases at E16 and E18, and is statistically significant (Wilcoxon rank-sum test *P* < 0.05) when compared to E14. Chicken *G6PDH* was used as a normalizer. **d**–**i** Type IV collagen protein and mRNA expression in the lung. **d**–**g** COL4A1 protein is found throughout the lung interstitium and epithelium (arrows) in the prenatal (E18.5) and postnatal (P6, P30) lungs but mainly in the interstitium at E16.5. At P6 and P30 murine COL4A1 protein is found at the tips of secondary septa (arrowhead in FGF). **h**–**k** *Col4a1* mRNA is almost undetectable by in situ hybridization at E16.5 (**h**) and is later found in a patchy distribution throughout the interstitium (arrows in **i**–**k**) with high expression at the tips of primary and secondary septa *(*arrowheads). Scale bar = 100 μm and applies to **d**–**i**

Additionally there are minor typographical errors stated below:

Additional file 3: Figure S3 has been revised: The image in the lefthand panel of the 3rd row of Additional file 3: Figure S3 (w/ y-axis labeled "PSPC PDPL") was wrong.

Page 4, second paragraph, third sentence: "week" should be "weak", and the figure panel mentioned last in that sentence should read, "(Fig. 1i-k), not g-i.

The correct sentence is below:

**"Col4a1mRNA expression was very weak at epithelial tips and surrounding mesenchyme at E16.5 (Fig.****1****h), but later, at E18.5, P6, and P30, it was clearly found in the lung interstitium and at the tips of the developing septa (Fig.****1****i-k)."**

Page 8, first paragraph, first sentence contains a split infinitive; it should read, "To show abnormalities more clearly in the development of type I pneumonocytes…".

Page 11, third paragraph, the word "defects" is missing, which alters the nuance, making this sentence confusing. The sentence should read: "The absence of branching defects may be because we analyzed heterozygous mutants in which sufficient levels of native protein…".

## Additional file

Additional file 3: Figure S3. (A–D) Histology of *R26-Cre* ^*ER*^ *; Col4a1* ^*+/Flex41*^ and *Tie2-Cre; Col4a1* ^*+/Flex41*^ mutants, and (E–I) immunohistochemistry analysis of PDPL-pSPC, HOPX, α-SMA, tropoelastin, and elastin in *R26-Cre* ^*ER*^ *; Col4a1* ^*+/Flex41*^. (A, B) Hematoxylin and eosin shows that both *R26-Cre* ^*ER*^ *; Col4a1* ^*+/Flex41*^ and *Tie2-Cre; Col4a1* ^*+/Flex41*^ mutants have simplified alveolarization. (C) *R26-Cre* ^*ER*^ *; Col4a1* ^*+/Flex41*^ septa are thick and with numerous blood capillaries (arrowheads) and cells with lipid content (arrows). (D) *Tie2-Cre; Col4a1* ^*+/Flex41*^ septa are small and short with increases in blood capillaries (arrowheads), but not in cells with lipid content. (E) pSPC and PDPL co-staining shows a disorganized alveolar epithelium. (F) *R26-Cre* ^*ER*^ *; Col4a1* ^*+/Flex41*^ display a decrease of type I pneumocytes as shown by nuclear staining of HOPX. (G–I) Abnormal localization of α-SMA, tropoelastin and elastin in the septa of *R26-Cre* ^*ER*^ *; Col4a1* ^*+/Flex41*^ lungs*.* Scale bars = 200 μm in A and B, 35 μm in C and D, 50 μm in E, and 100 μm in F to I. (TIF 24706 kb)
