# Supplementary figures and images for: Type IV collagen drives alveolar epithelial–endothelial association and the morphogenetic movements of septation
Source: BMC Biol. 2016 Jul 13;14:59. doi: 10.1186/s12915-016-0281-2 (PMC4942891; doi:10.1186/s12915-016-0281-2)

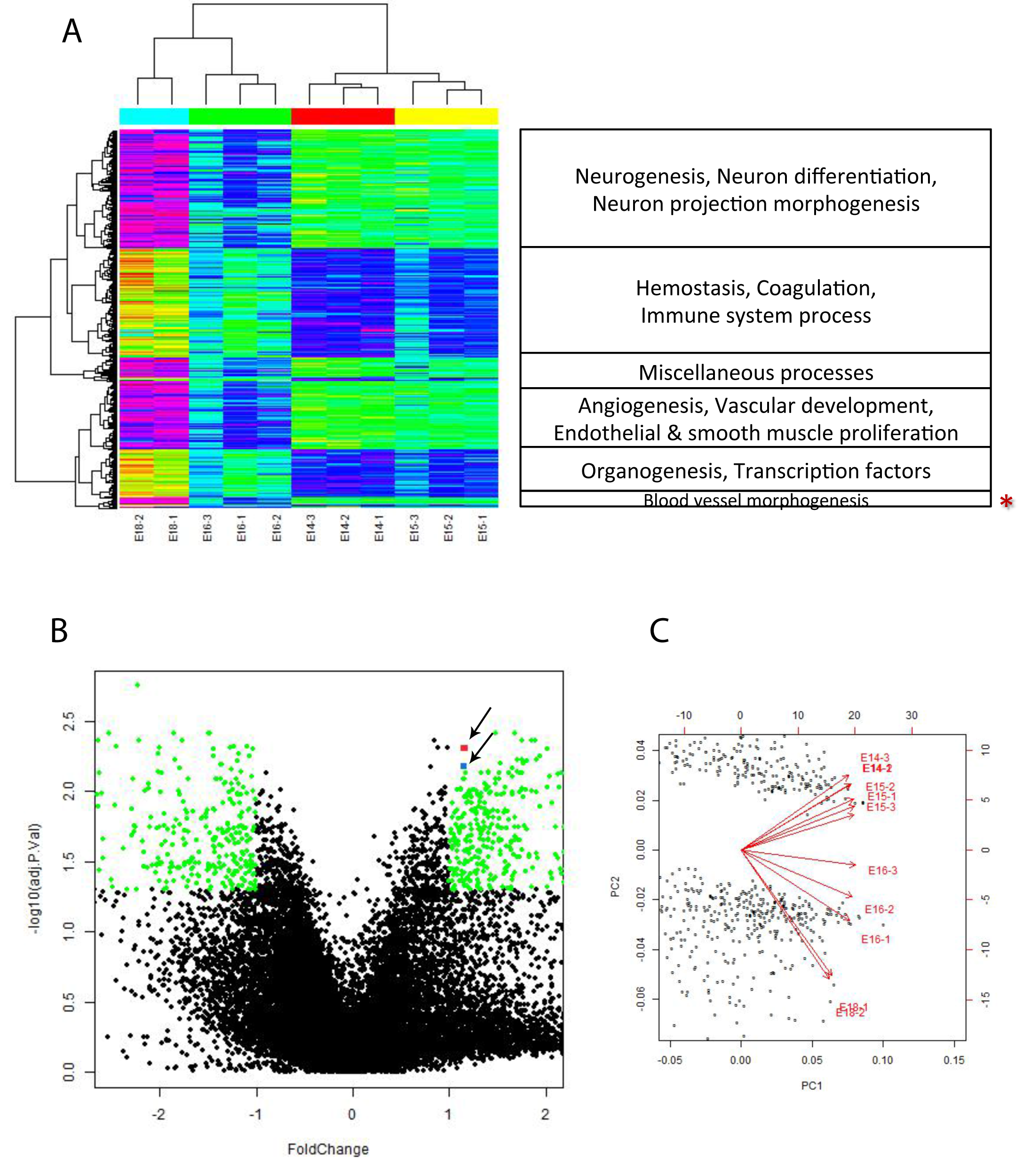

Supplement: Additional file 1: Figure S1. — Heatmap and volcano plot of microarray data. (A) Heatmap of differentially expressed genes across the four time points. Samples cluster according to time point (blue: E18, green: E16, red: E14 and yellow: E15). The unsupervised hierarchical clustering of all the transcripts forms six discrete groups. From top to bottom, cluster A (n = 190 chick transcripts) contains genes involved in neurogenesis, neuron differentiation and neuron projection morphogenesis; cluster B (n = 206) is enriched for hemostasis, coagulation and immune system process genes; cluster C (n = 42) contains various genes involved in miscellaneous processes; cluster D (n = 119) is characterized by angiogenesis, vascular development, endothelial and smooth muscle proliferation genes; cluster E (n = 84) groups genes, mostly transcription factors, with a role in organogenesis. Finally, cluster F (n = 18, indicated by the red asterisk) contains a limited number of genes involved in blood vessel morphogenesis and includes Adam12, Col4a1, Col4a2, Crmp1, Emp1, Epas1, Fbln5, Meis1, Mgp, Podxl, Snai2, and Wnt11. GO enrichment analyses were computed by the Panther Classification System, available at http://geneontology.org. GO annotations were retrieved after one-to-one conversion of chick to mouse orthologs by Ensembl Biomart, accessible at http://www.ensembl.org/biomart/martview. (B) A volcano plot was used to compare fold change from E14 to E18 of log2 normalized data (x-axis) and –log10 of the adjusted P value across the four time points, computed by limma. Transcripts with adjusted P values less than or equal to 0.05 and fold change greater than, or equal to, 1 are shown in green. The Col4a1 and Col4a2 transcripts are indicated by the red and blue squares (top right), respectively. (C) Bi-plot of the Principal Component Analysis results, conducted using the prcomp function to identify potential sample outliers. (TIF 16074 kb) [file 12915_2016_281_MOESM1_ESM.tif]

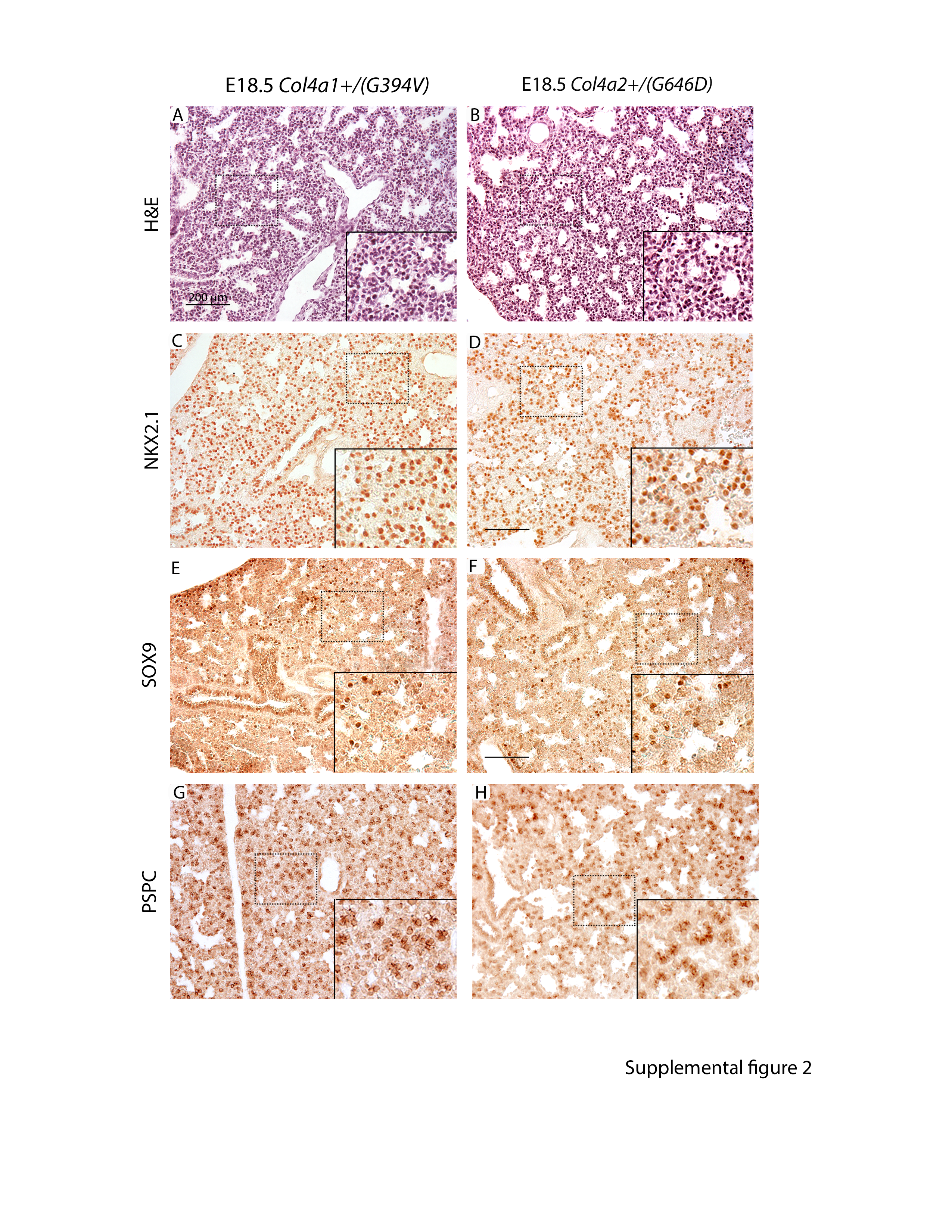

Supplement: Additional file 2: Figure S2. — Histology and immunohistochemistry analyses of the distal epithelium of Col4a1 +/G394V and Col4a2 +/G646D mutants. (A, B) Hematoxylin and eosin staining shows thickened interstitium and the small primitive alveolar sacs in Col4a1 and Col4a2 mutant lungs. Immunohistochemistry of NKX2.1 (C, D), SOX9 (E, F) and PSPC (F, G) in Col4a1 +/G394V and Col4a2 +/G646D display clusters of the distal epithelium. Scale bars = 200 μm in A to G. (TIF 24701 kb) [file 12915_2016_281_MOESM2_ESM.tif]

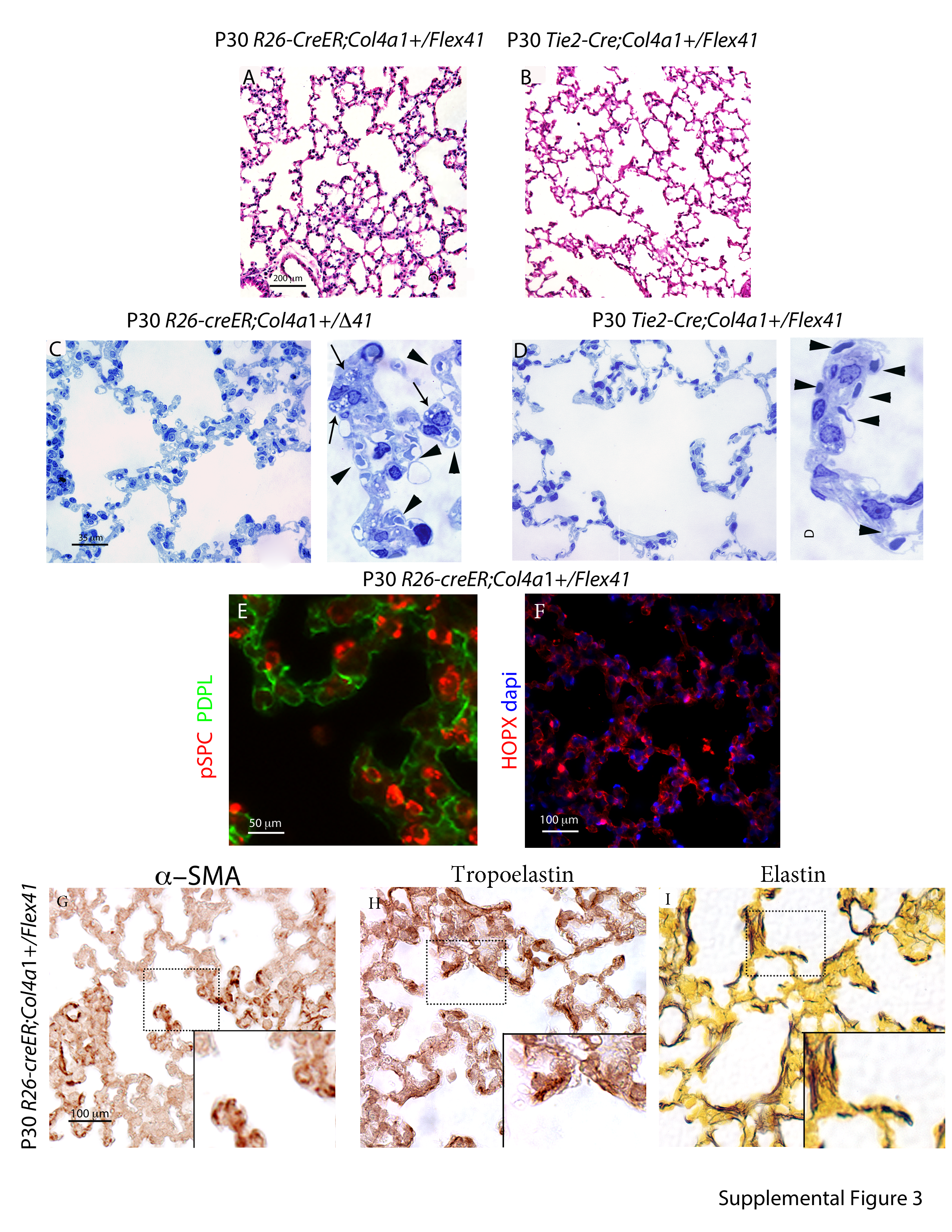

Supplement: Additional file 3: Figure S3. — (A–D) Histology of R26-Cre ER ; Col4a1 +/Flex41 and Tie2-Cre; Col4a1 +/Flex41 mutants, and (E–I) immunohistochemistry analysis of PDPL-pSPC, HOPX, α-SMA, tropoelastin, and elastin in R26-Cre ER ; Col4a1 +/Flex41. (A, B) Hematoxylin and eosin shows that both R26-Cre ER ; Col4a1 +/Flex41 and Tie2-Cre; Col4a1 +/Flex41 mutants have simplified alveolarization. (C) R26-Cre ER ; Col4a1 +/Flex41 septa are thick and with numerous blood capillaries (arrowheads) and cells with lipid content (arrows). (D) Tie2-Cre; Col4a1 +/Flex41 septa are small and short with increases in blood capillaries (arrowheads), but not in cells with lipid content. (E) pSPC and PDPL co-staining shows a disorganized alveolar epithelium. (F) R26-Cre ER ; Col4a1 +/Flex41 display a decrease of type I pneumocytes as shown by nuclear staining of HOPX. (G–I) Abnormal localization of α-SMA, tropoelastin and elastin in the septa of R26-Cre ER ; Col4a1 +/Flex41 lungs. Scale bars = 200 μm in A and B, 35 μm in C and D, 50 μm in E, and 100 μm in F to I. (TIF 24706 kb) [file 12915_2016_281_MOESM3_ESM.tif]

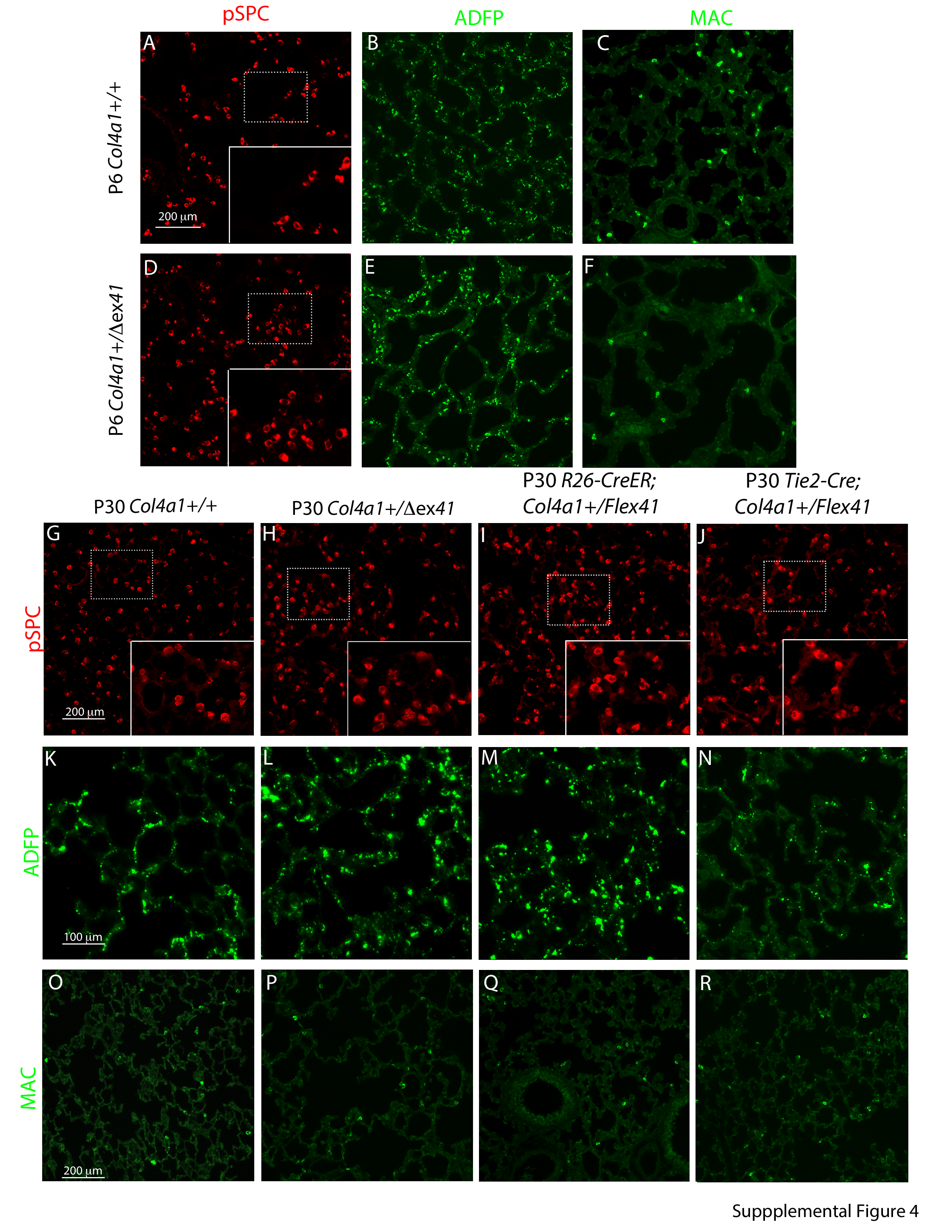

Supplement: Additional file 4: Figure S4. — Immunofluorescence of pSPC, ADFP and MAC in Col4a1 +/∆ex41, R26-Cre ER ; Col4a1 +/Flex41, and Tie2-Cre; Col4a1 +/Flex41. (A, D, G–J) pSPC normal localization at P6 (A) and P30 (G) is spread throughout the lung alveoli, while in P6 and P30 Col4a1 +/Flex41 (D, H) and in P30 R26-Cre ER ; Col4a1 +/Flex41 (I) it is patchy and a slightly increased. This patchy Col4a1 +/∆ex41 and R26-Cre ER ; Col4a1 +/Flex41 distribution of pSPC is not observed in Tie2-Cre; Col4a1 +/Flex41 (J). (E, L, M) Lipofibroblast staining with ADFP marker shows increased of ADFP+ cells in P6 and P30 Col4a1 +/∆ex41 (E, L) and in P30 R26-Cre ER ; Col4a1 +/Flex41 (M) lungs compared with normal lungs (B, K). (N) Tie2-Cre; Col4a1 +/Flex41 displays decreased ADFP+ cells. (C, F) Decrease of MAC+ cells in Col4a1 +/∆ex41 (F) at P6, but not as clear at P30 (O–R). Scale bars = 200 μm in A to J and O to R, 100 μm in K to N. (TIF 24708 kb) [file 12915_2016_281_MOESM4_ESM.tif]

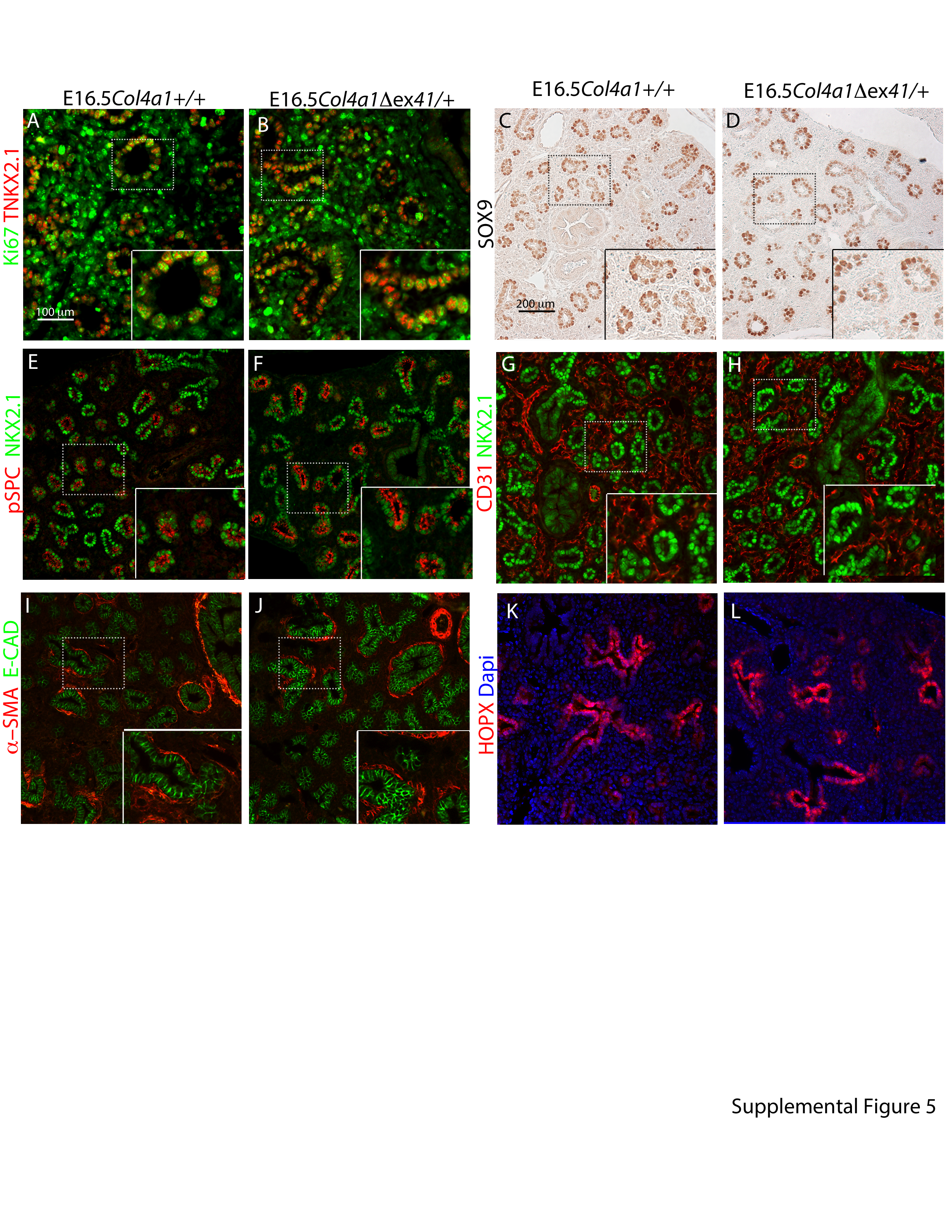

Supplement: Additional file 5: Figure S5. — (A–F) Immunohistochemistry analyses of E16.5 Col4a1 +/Δex41. (A, B) Double immunofluorescence Ki67 and NKX2.1 in Col4a1 +/Δex41 and wild type lungs show no differences in overall proliferation. Mutants display no differences in SOX9 (C, D), pSPC and NKX2.1 (E, F), CD31 and NKX2.1 (G, H), α-SMA and E-CAD (I, J), or HOPX (K, L) localization. Scale bars = 100 μm in A and B, 200 μm in C to L. (TIF 24679 kb) [file 12915_2016_281_MOESM5_ESM.tif]

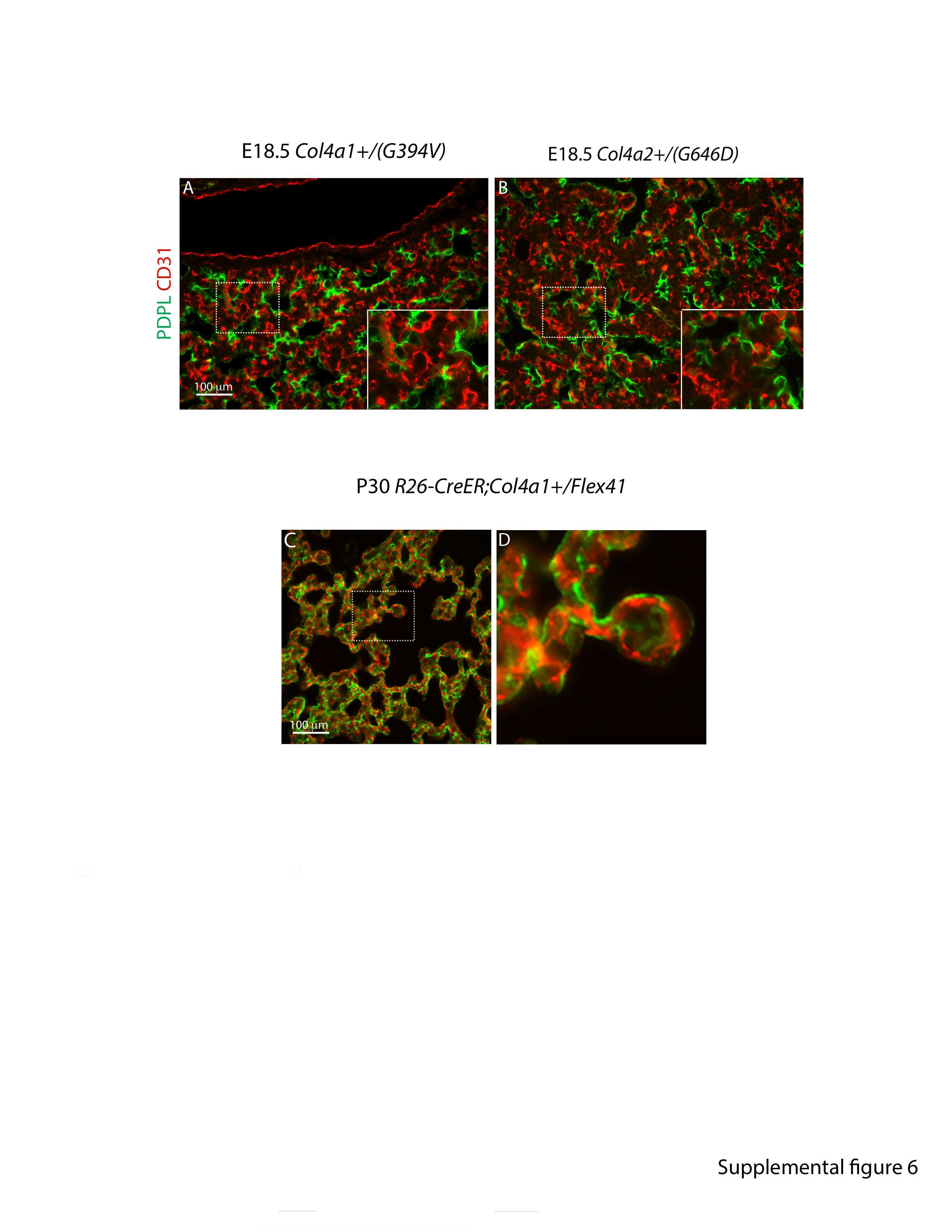

Supplement: Additional file 6: Figure S6. — Immunofluorescence of PDPL and CD31 Col4a1 +/G394V, Col4a2 +/G646D mutants, and R26-Cre ER ; Col4a1 +/Flex41. (A, B) PDPL and CD31 staining shows vascular disorganization around collapse saccules in Col4a1 +/G394V (A) and Col4a2 +/G646D (B). This disorganization is also observed in P30 R26-Cre ER ; Col4a1 +/Flex41 (C, D). Scale bars = 100 μm in A and B, 200 μm in C. (TIF 24703 kb) [file 12915_2016_281_MOESM6_ESM.tif]

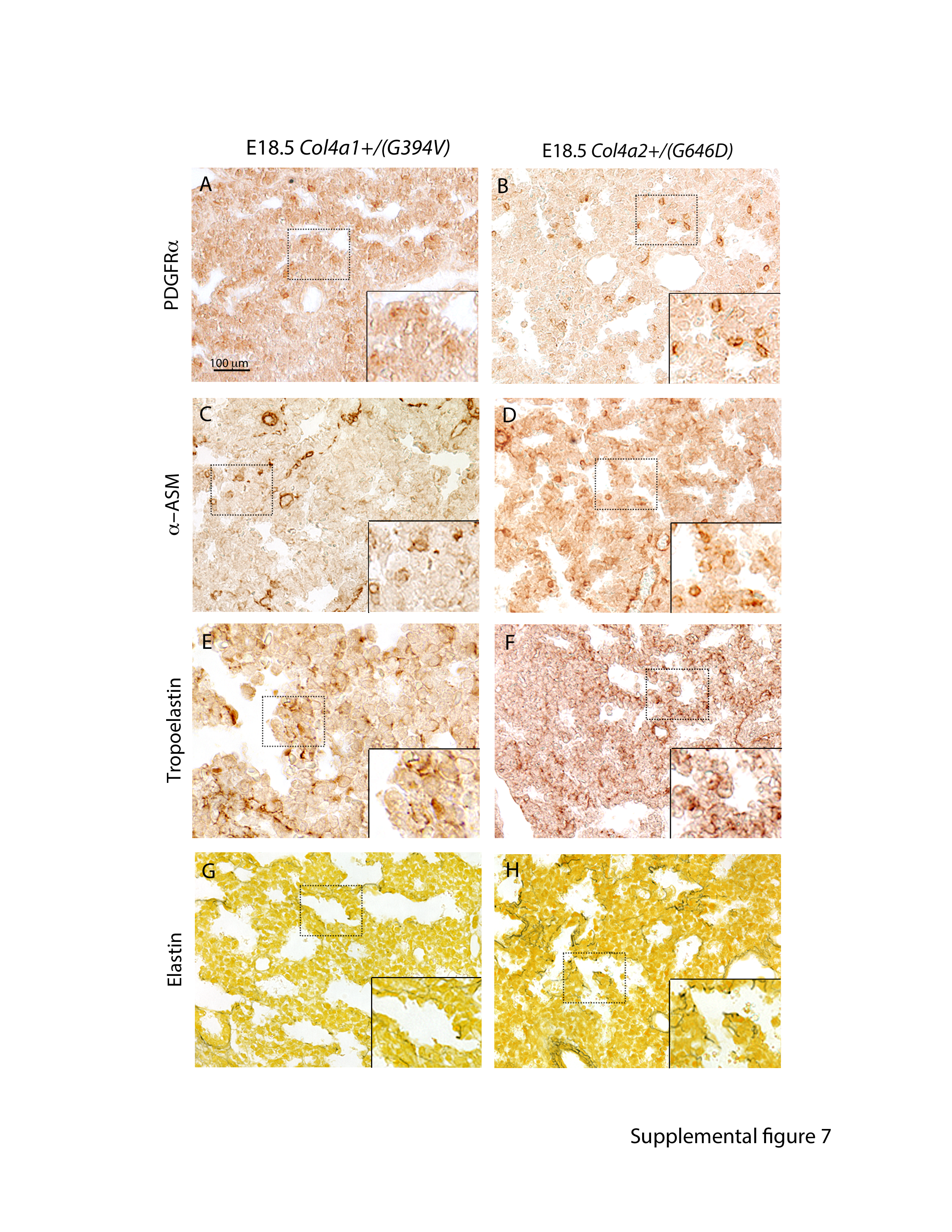

Supplement: Additional file 7: Figure S7. — Immunohistochemistry of PDGFRα, α-SMA, tropoelastin, and elastin in Col4a1 +/G394V and Col4a2 +/G646D lungs. Col4a1 and Col4a2 mutant lungs exhibit a decreased and patchy distribution of PDGFRα (A, B), SMA-α (C, D), and tropoelastin expression (E, F). Elastin fibers are atypical and decreased in Col4a1 +/G394V (G) and Col4a2 +/G646D (H) lungs. Scale bars = 100 μm in A to H. (TIF 24703 kb) [file 12915_2016_281_MOESM7_ESM.tif]

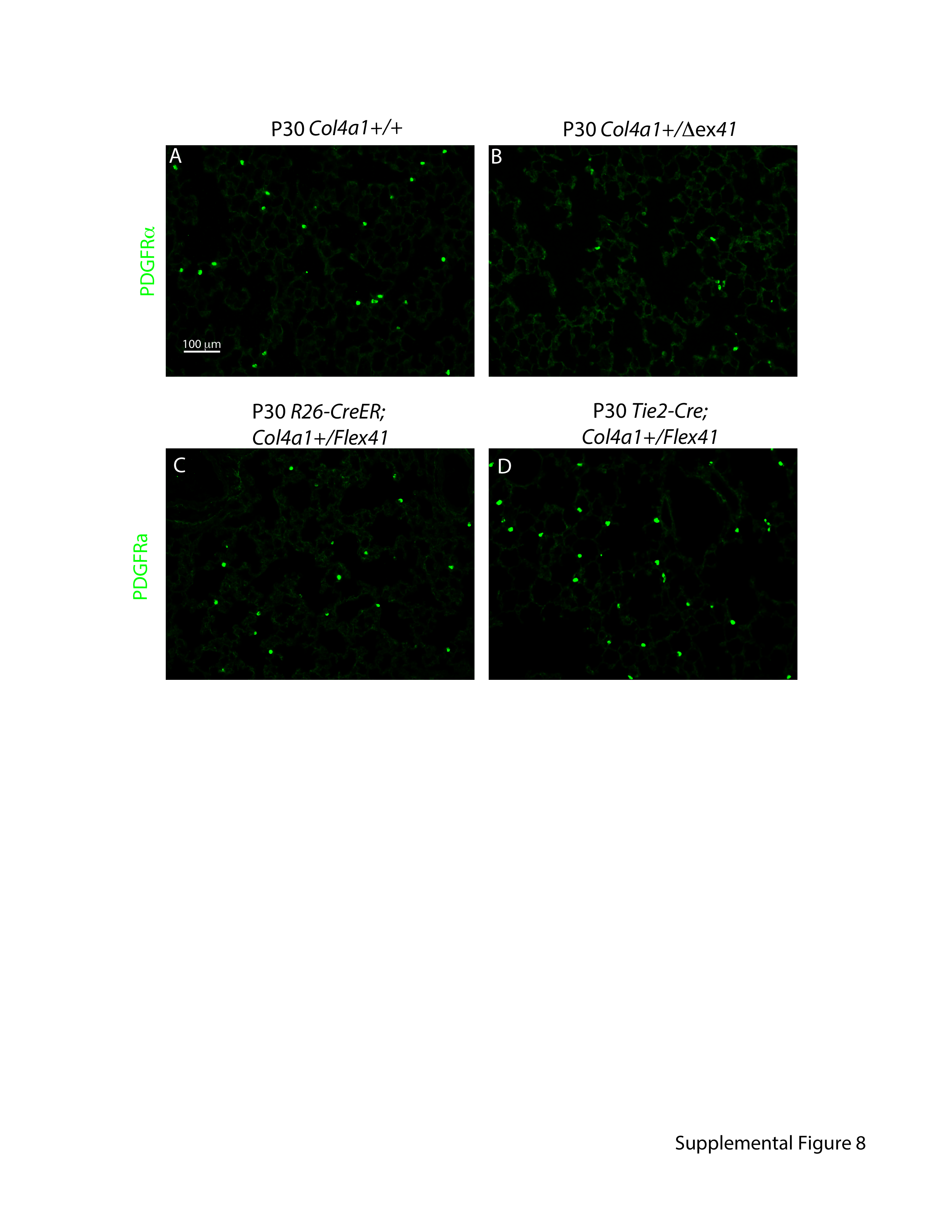

Supplement: Additional file 8: Figure S8. — PDGFRα localization in P30 mutant lungs. (A–D) At P30, Col4a1 +/Δex41 (B) has fewer PDGFRα+ cells compared to normal (A), R26-Cre ER ; Col4a1 +/Flex41 (C) and Tie2-Cre; Col4a1 +/Flex41 (D) lungs. Scale bars = 100 μm in A to D. (TIF 24700 kb) [file 12915_2016_281_MOESM8_ESM.tif]

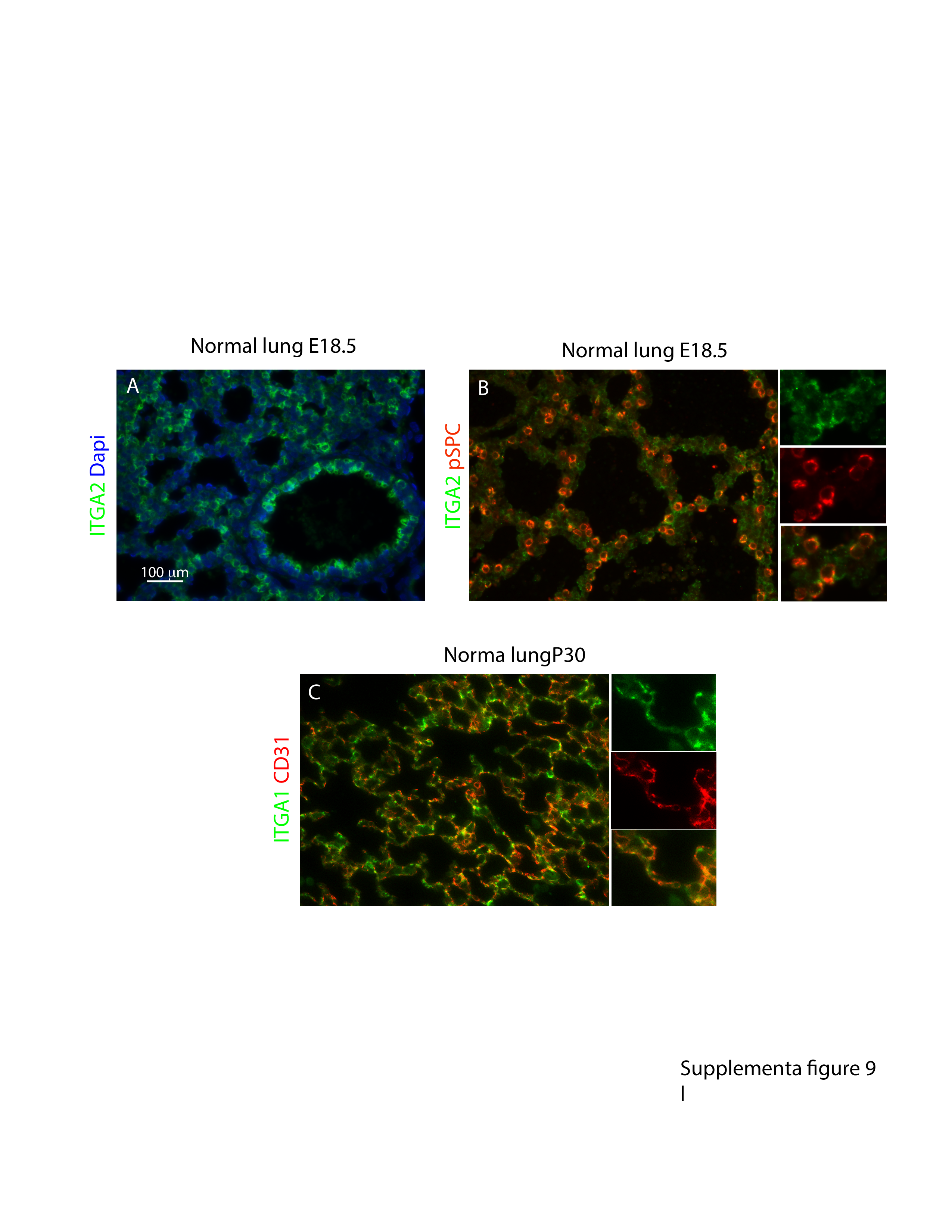

Supplement: Additional file 9: Figure S9. — (A, C) ITGA2 and ITGA1 localization in normal lungs. (A–C) During development, at E18.5, ITGA2 displays epithelial localization that partially co-localizes with pSPC (A, B). At P30, ITGA1 expression is co-stained with CD31, marking the vasculature (C). Scale bars = 100 μm in A–C. (TIF 24700 kb) [file 12915_2016_281_MOESM9_ESM.tif]
