# Supplementary figures and images for: Erratum to: Type IV collagen drives alveolar epithelial–endothelial association and the morphogenetic movements of septation
Source: BMC Biol. 2016 Sep 1;14(1):73. doi: 10.1186/s12915-016-0297-7 (PMC5009649; doi:10.1186/s12915-016-0297-7)

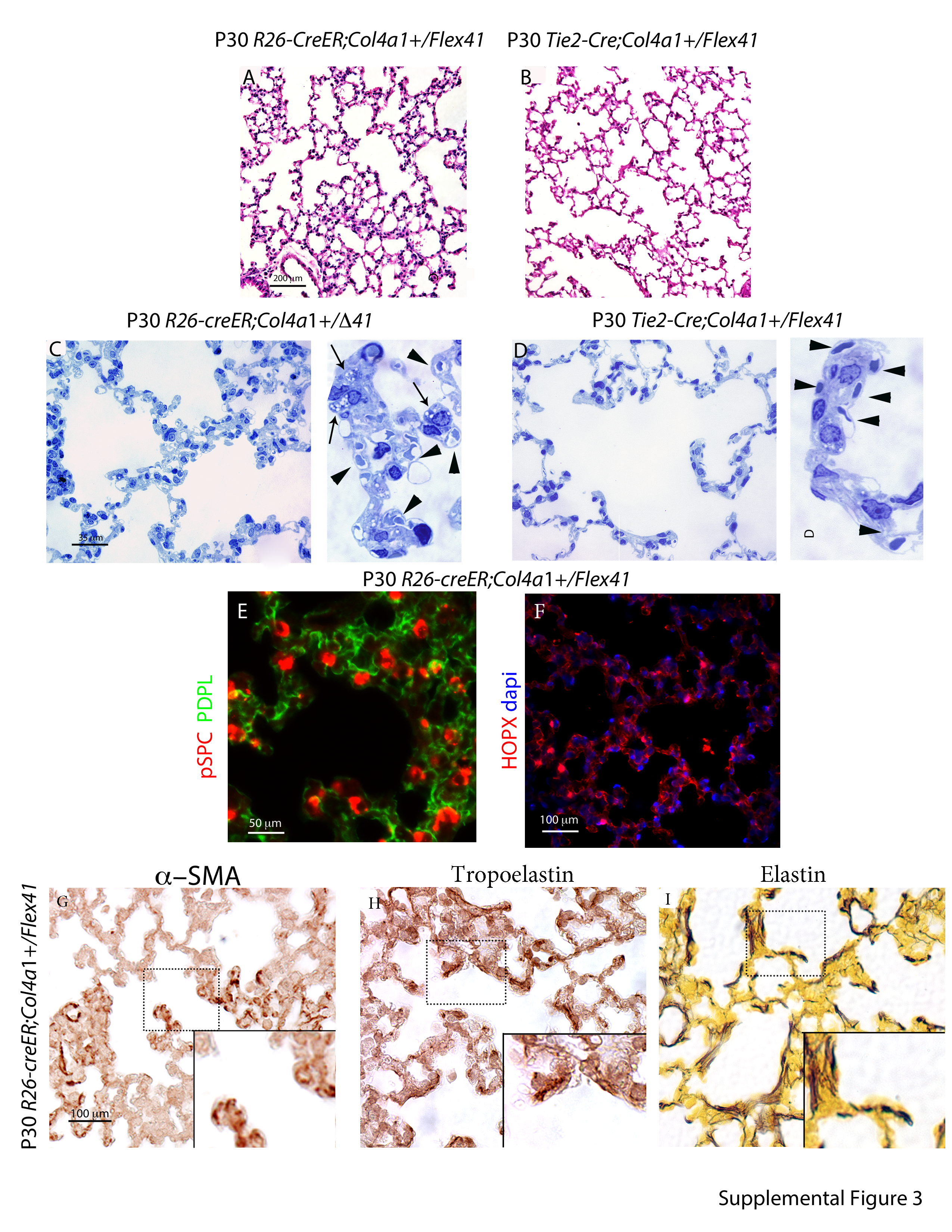

Supplement: Additional file 3: Figure S3. — (A–D) Histology of R26-Cre ER ; Col4a1 +/Flex41 and Tie2-Cre; Col4a1 +/Flex41 mutants, and (E–I) immunohistochemistry analysis of PDPL-pSPC, HOPX, α-SMA, tropoelastin, and elastin in R26-Cre ER ; Col4a1 +/Flex41. (A, B) Hematoxylin and eosin shows that both R26-Cre ER ; Col4a1 +/Flex41 and Tie2-Cre; Col4a1 +/Flex41 mutants have simplified alveolarization. (C) R26-Cre ER ; Col4a1 +/Flex41 septa are thick and with numerous blood capillaries (arrowheads) and cells with lipid content (arrows). (D) Tie2-Cre; Col4a1 +/Flex41 septa are small and short with increases in blood capillaries (arrowheads), but not in cells with lipid content. (E) pSPC and PDPL co-staining shows a disorganized alveolar epithelium. (F) R26-Cre ER ; Col4a1 +/Flex41 display a decrease of type I pneumocytes as shown by nuclear staining of HOPX. (G–I) Abnormal localization of α-SMA, tropoelastin and elastin in the septa of R26-Cre ER ; Col4a1 +/Flex41 lungs. Scale bars = 200 μm in A and B, 35 μm in C and D, 50 μm in E, and 100 μm in F to I. (TIF 24706 kb) [file 12915_2016_297_MOESM3_ESM.tif]
